# A comprehensive study of the genomic differentiation between temperate Dent and Flint maize

**DOI:** 10.1186/s13059-016-1009-x

**Published:** 2016-07-08

**Authors:** Sandra Unterseer, Saurabh D. Pophaly, Regina Peis, Peter Westermeier, Manfred Mayer, Michael A. Seidel, Georg Haberer, Klaus F. X. Mayer, Bernardo Ordas, Hubert Pausch, Aurélien Tellier, Eva Bauer, Chris-Carolin Schön

**Affiliations:** Plant Breeding, TUM School of Life Sciences Weihenstephan, Technical University of Munich, 85354 Freising, Germany; Section of Population Genetics, TUM School of Life Sciences Weihenstephan, Technical University of Munich, 85354 Freising, Germany; Present Address: Institute for Crop Science and Plant Breeding, Bavarian State Research Center, 85354 Freising, Germany; Plant Genome and System Biology, Helmholtz Zentrum München, 85764 Neuherberg, Germany; Misión Biológica de Galicia, Spanish National Research Council (CSIC), 36080 Pontevedra, Spain; Animal Breeding, TUM School of Life Sciences Weihenstephan, Technical University of Munich, 85354 Freising, Germany

**Keywords:** Maize, Flint, Dent, Selection, Population genetics, Genomics, Genome-wide screen, Landraces

## Abstract

**Background:**

Dent and Flint represent two major germplasm pools exploited in maize breeding. Several traits differentiate the two pools, like cold tolerance, early vigor, and flowering time. A comparative investigation of their genomic architecture relevant for quantitative trait expression has not been reported so far. Understanding the genomic differences between germplasm pools may contribute to a better understanding of the complementarity in heterotic patterns exploited in hybrid breeding and of mechanisms involved in adaptation to different environments.

**Results:**

We perform whole-genome screens for signatures of selection specific to temperate Dent and Flint maize by comparing high-density genotyping data of 70 American and European Dent and 66 European Flint inbred lines. We find 2.2 % and 1.4 % of the genes are under selective pressure, respectively, and identify candidate genes associated with agronomic traits known to differ between the two pools. Taking flowering time as an example for the differentiation between Dent and Flint, we investigate candidate genes involved in the flowering network by phenotypic analyses in a Dent–Flint introgression library and find that the Flint haplotypes of the candidates promote earlier flowering. Within the flowering network, the majority of Flint candidates are associated with endogenous pathways in contrast to Dent candidate genes, which are mainly involved in response to environmental factors like light and photoperiod. The diversity patterns of the candidates in a unique panel of more than 900 individuals from 38 European landraces indicate a major contribution of landraces from France, Germany, and Spain to the candidate gene diversity of the Flint elite lines.

**Conclusions:**

In this study, we report the investigation of pool-specific differences between temperate Dent and Flint on a genome-wide scale. The identified candidate genes represent a promising source for the functional investigation of pool-specific haplotypes in different genetic backgrounds and for the evaluation of their potential for future crop improvement like the adaptation to specific environments.

**Electronic supplementary material:**

The online version of this article (doi:10.1186/s13059-016-1009-x) contains supplementary material, which is available to authorized users.

## Background

**Fig. 1:**
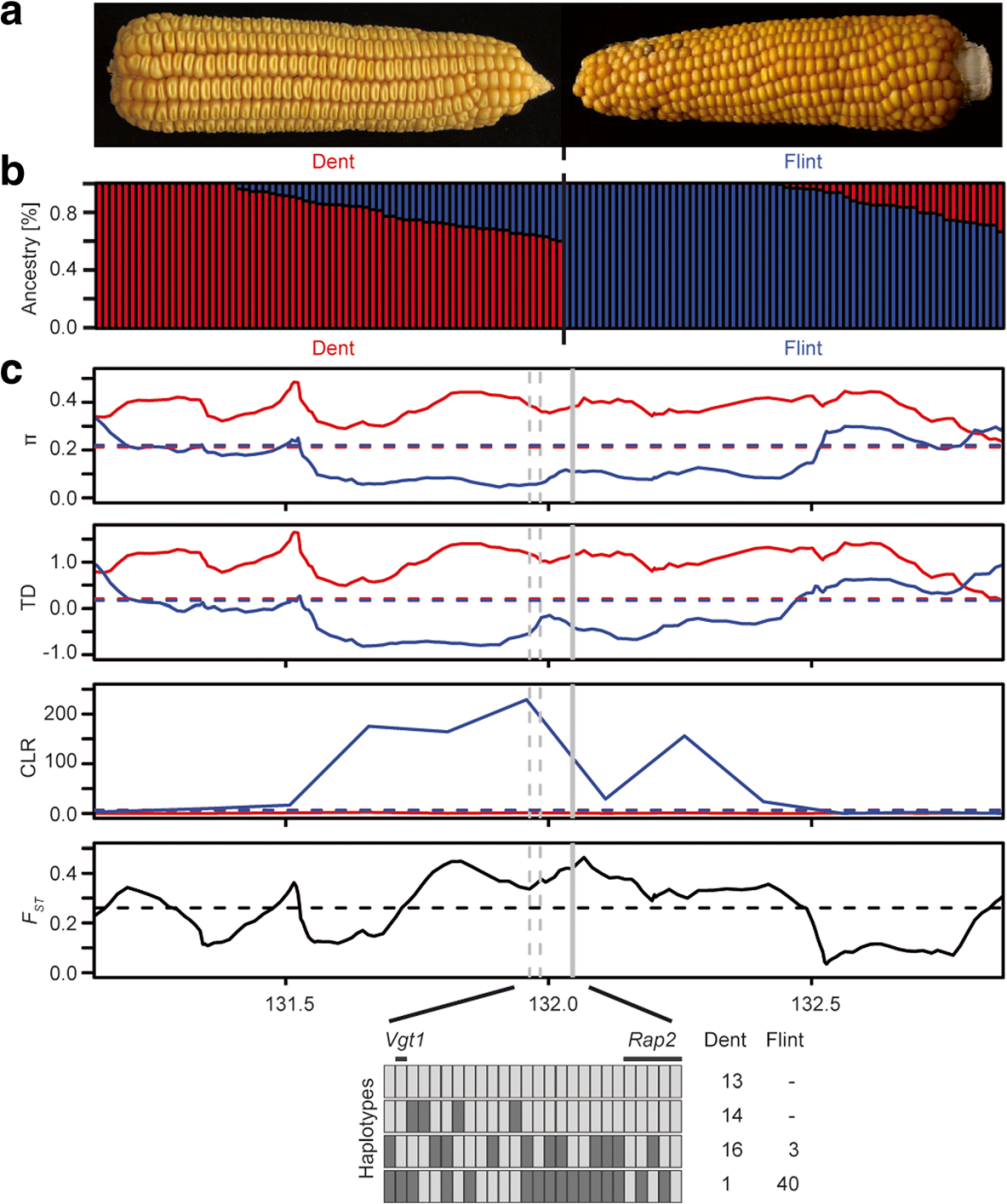
Population structure of the investigated 136 Dent and Flint elite lines and detection of pool-specific selection signatures. **a** *Images* of maize cobs with Dent-type (*left*) and Flint-type kernels (*right*) as an example for phenotypic differences between the two germplasm pools. **b** Population structure and assignment of 136 temperate maize elite lines to Dent (*red*; N = 70) and Flint (*blue*; N = 66) pools. *Bar plots* indicate the relative ancestral composition of the lines. **c** Sweep statistics based on the panel of 136 temperate inbred lines shown exemplarily for a region on chromosome 8 that includes the *Vgt1* locus (*dashed gray lines*) and *Rap2* (*solid gray line*). Within-group statistics (*π*, TD, and CLR) are shown in *red* for Dent and in *blue* for Flint. *Horizontal dashed lines* indicate the cutoff per statistic (10 % quantile for *π* and TD, 90 % quantile for CLR and *F*_*ST*_). For the region encompassed by the two loci *Vgt1* and *Rap2*, the four major haplotypes observed in the panel are shown. *Light gray boxes* indicate the B73 reference allele and *dark gray boxes* the alternative allele of each SNP. *Numbers on the right side* of the haplotype plot refer to the number of observations per haplotype within the Dent and the Flint panels

Maize is one of the world’s major staple crops but considerable concern is arising that ongoing anthropogenic global warming will have drastic effects on maize production and might result in a reduction of up to 10 % in yield in the near future [1]. Expanding production areas to higher latitudes could moderate the effect, but this would require the adaptation of breeding material to shorter vegetation periods. Breeders can cope with this challenge by taking advantage of the tremendous genetic diversity of maize that is available in different temperate breeding pools. Two of the major pools exploited in breeding are the Dent and Flint germplasm pools with their names referring to different kernel phenotypes [2]. Dents have characteristic indented kernels with high soft starch content, whereas Flints have kernels with a thick, hard, and vitreous outer layer (Fig. 1a). The genetic divergence of these two pools can be explained by their historic geographical separation [3] and adaptation to different environments. Among all maize germplasm, Northern Flints reached the highest latitudes like the northern regions of the U.S. and Canada, which required selection for early maturity and cold tolerance [3]. These Northern Flints, together with Caribbean germplasm, were major progenitors of European maize and enabled the rapid adaptation to European climates [4]. Especially in cooler regions of Europe, breeding programs exploit heterotic effects between Dent lines tracing back to U.S. Corn Belt Dents and Flint lines, with Flint contributing early vigor and good cold tolerance and Dent contributing high productivity to the hybrids. The divergence of the Dent and Flint germplasm pools has been described in diversity studies based on molecular markers [5] and also in genetic studies mapping quantitative trait loci (QTL) underlying agronomic traits. A recent study utilizing Dent and Flint nested association mapping (NAM) populations [6] found little overlap of QTL for five complex traits between the two pools [7]. Although QTL mapping is a useful tool to elucidate the genetic architecture of phenotypic traits, it can only unravel genomic regions for which the genetic material under study is segregating, whereas regions under selection can be missed in case of near or complete fixation. Thus, alternative approaches are needed to investigate the divergence of Dent and Flint on a genomic level and to further elucidate how selection shaped the pool-specific genomic diversity.

Selection creates specific patterns of diversity in the genome [8] and these signatures can be used for the detection of regions under selection. When a favorable, new (derived compared to the ancestral) allele rises in frequency within a population, selective sweeps are generated, which are characterized by a local reduction in nucleotide diversity and high derived allele frequencies [9–11]. In addition, strong and recent sweeps will display large blocks with high linkage disequilibrium surrounding the derived mutation as the dispersal of the new allele will be faster than recombination is able to break down linkage disequilibrium [12, 13]. The identification of selection signatures through genome-wide screens provides an efficient way to detect selection candidates and methods for their detection are often combined to reduce the number of false-positives [14–16]. In maize, genome-wide screens for selection signatures were successfully applied to identify genes involved in domestication and improvement and allowed insights into evolutionary processes shaping the genome diversity of maize [17–20]. Taking advantage of the characteristics of selective sweeps and using high-density genotyping data from a maize 600 k single nucleotide polymorphism (SNP) array [21], we screened a panel of 136 temperate Dent and Flint elite lines for extreme allele frequencies over extended linked sites to identify genomic regions under selective pressure and to gain insights into the genomic variation underlying the differentiation of Dent and Flint. We included outgroup information from *Sorghum bicolor* to further support the identified candidate genes based on derived allele frequencies. We furthermore investigated the candidate genes based on whole-genome sequence data of 40 Dent and Flint lines [21, 22] and examined if genic and upstream regions contributed equally to the differentiation between temperate Dent and Flint.

The elite line panel under study comprised frequently used and important founder lines exploited in breeding programs for temperate climates. The Dent lines in our panel represent U.S. Corn Belt and European material, whereas most of the Flint lines originated from European breeding programs. Based on the selection screens, we examined pool-specific enrichment of candidate genes for metabolic pathways and investigated candidates associated with traits that are known to differentiate Dent and Flint like cold tolerance and flowering time [23, 24]. Flowering time is essential for local adaptation and represents a major determinant for other agronomic traits, such as grain filling and yield. The complex genetic architecture of flowering time has been studied in maize in a large number of studies mapping QTL with a meta-QTL analysis revealing 62 flowering time consensus QTL [25]. Phenotypic differences in maize flowering time are mainly caused by the accumulation of many small-effect QTL [26] and only a few large-effect genes have been characterized so far [27–30]. Hundreds of homologs to *A. thaliana* flowering time genes have been found in the maize genome [31], but in most cases their functional roles in the maize flowering network remain to be elucidated [25, 26, 29, 32–37]. In this study, we identified candidate genes from the flowering network with haplotypes near fixation or fixed in either of the two elite pools. We used this set of genes as an example to characterize genomic differentiation between Dent and Flint in more detail. We evaluated the effect of these genes on flowering time in a Dent–Flint introgression library and investigated their assignment to different pathways within the flowering network. To assess the congruency of the allelic composition of the candidate genes between elite lines and landraces, we expanded our candidate gene analysis to a large dataset of 38 European landraces that comprises more than 900 individuals. By exploring this unique resource, we gained insights into the genetic variation of the selection candidates between landraces and elite lines and investigated, which landraces likely contributed to the observed candidate gene diversity in the elite lines and if haplotypes not yet exploited in breeding could be detected. Taken together, our study allowed insights into patterns of differentiation between temperate Dent and Flint germplasm and provided candidates for follow-up studies to characterize their biological and molecular functions, to investigate their impact on phenotypes, and to assess their potential use for further crop improvement.

## Results and discussion

### Characterization of the Dent and Flint panels

We genotyped a diverse panel of 136 temperate inbred lines (Additional file 1: Table S1) at high density with the Axiom® Maize Genotyping Array [21]. The array comprises more than 600 k SNP markers, which were identified based on mid- to high-coverage whole-genome sequence data of 30 representative temperate Dent and Flint maize lines [21]. Markers were filtered according to quality scores and stable performance on the array, thus representing high-confidence sequence variants, and their final distribution followed the average recombination rate along the chromosomes [21]. After stringent quality filtering of the 616,201 markers included on the array, 547,412 high-quality SNPs (88.8 %) remained for analysis. These SNPs tagged 19,759 genes (49.8 % of the annotated gene set of maize) with, on average, two SNPs in their coding region (52.6 % synonymous and 47.4 % non-synonymous). Slightly more SNPs were polymorphic in the Flint compared to the Dent panel (95.4 % versus 93.1 %), but the majority of SNPs segregated in both germplasm pools (88.6 %).

The panel of 136 temperate Dent and Flint inbred lines comprised frequently used and important founder lines exploited in breeding programs in Europe and the U.S., including lines which were used as parents for the U.S. and European NAM panels [6, 38, 39]. The 70 Dent lines were selected according to available pedigree information and their frequency of use and citation [40, 41] to assemble a representative set of lines. Besides 16 European Dent lines, the lines represent U.S. Corn Belt Dent and include lines from the Maize Association Population [42] and the list of inbred lines with expired U.S. plant variety protection [43]. The 66 Flint lines investigated in this study comprised important founder lines of European breeding programs like F2 and F7 originating from the French landrace Lacaune, EP1 from the Spanish landrace Lizargarate, and derivatives of the German landrace Gelber Badischer Landmais [44]. The Flints comprised in total 34 lines from France, 20 from Germany, four from Spain, three from Italy, three from North America, as well as one from Switzerland and Austria. Between the elite lines of the two germplasm pools, we observed a clear separation of pools (Fig. 1b) and a high genome-wide level of differentiation (*F*_*ST*_ = 0.14), which is consistent with the long-term genetic differentiation between Dent-type and Flint-type maize [2, 3].

### Genome-wide screens for selection signals

Taking advantage of the characteristics of selective sweeps, we screened the genome for extreme allele frequencies over extended linked sites to detect regions under differential selective pressure between Dent and Flint. Signatures of selection in only one of the two pools, Dent or Flint, were detected based on low levels of nucleotide diversity (*π*) [9] and Tajima’s *D* (TD) [10] in the respective pool. In addition, a signature had to be supported by a high value of the composite likelihood ratio (CLR) test [11] within the respective pool, which indicates a deviation of the allelic composition of a genetic region compared to a neutrally evolving sequence determined by the genomic background. To ensure that the selection signature was specific for one of the two pools, it had to be associated with a high level of differentiation between Dent and Flint measured by the fixation index *F*_*ST*_ [45]. Except for the CLR statistic, which was calculated for non-overlapping grids of 150 kb, we applied a sliding window approach averaging data over windows of 40 SNPs (sliding by 10 %) and filtered for regions below the 10 % quantile for *π* and TD and above the 90 % quantile for *F*_*ST*_ and CLR (Additional file 1: Table S2). Following the approach reported by [17], adjacent windows passing the threshold for all four statistics were grouped together for candidate gene analysis, as the observed changes in allele frequency were likely caused by the same selective sweep event. This resulted in a filtered set of 265 windows for Dent and 158 windows for Flint, with an average length of 331.40 kb and 267.80 kb, respectively, and thus comparable to the length of domestication windows found in a previous study [17]. An example of a signature of differential selection in Dent and Flint determined by all four metrics (*π*, TD, CLR, and *F*_*ST*_) is shown in Fig. 1c for a region on chromosome 8 harboring two candidate genes. The underlying genetic region was composed of four major haplotypes. The first three haplotypes occurred at intermediate frequencies in Dent, whereas the fourth haplotype was almost exclusive for Flint.

Genome-wide patterns of diversity and the resulting distribution of selection signatures in the Dent and Flint panels are given in Additional file 2: Figure S1. Within the filtered set of windows, which covered 4.3 % of the total length of the maize genome for Dent and 2.1 % for Flint, we identified 876 genes as candidates under differential selective pressure in Dent and 545 genes for Flint with 14 genes common to both candidate genes sets (Additional file 3: Table S3). This corresponded to 2.2 % and 1.4 % of the filtered gene set of maize, respectively, and is in the same order of magnitude as the estimated number of genes under selective pressure during maize domestication and improvement [17]. When comparing the candidate gene sets with the 571 improvement candidates reported by [17], 26 genes overlapped with the list of Dent candidates but only one gene with the Flint candidate gene set. Considering that the genetic material studied in [17] comprised mainly U.S. Dent and (sub-) tropical lines and that pool-specific sequence variation in temperate Dent and Flint has been reported here and, for example, by [5], these results emphasize the relevance of a representative panel of lines belonging to divergent germplasm pools to obtain a comprehensive picture of the genomic diversity in maize.

In genome-wide screens for signatures of positive selection, also other forces than selection, such as heterogeneous mutation and recombination rates along the genome, past demographic history and background selection shape the genomic diversity and can give rise to false-positive signals. It is beyond the scope of this paper to infer a full demographic history of maize for the elite lines and landraces as the breeding history of maize is complex and violates several assumptions of the classic population genetics models (e.g. discussed in [46]), as, for example, the assumption of panmictic populations and applicability of the coalescent at short time scales. We therefore applied the CLR test [11], which detects selective sweeps based on the comparison of the site-frequency spectrum within a specific genomic region to the average site-frequency spectrum over the genome, a method which has been successfully used in human and other species to detect selective sweeps [11, 47, 48]. To further decrease the rate of false-positives, the CLR test was combined with three additional metrics (*π*, TD, and *F*_*ST*_) and we identified signatures of positive selection based on this conservative approach with an overlap of genome-wide extreme values per metric. The high level of linkage disequilibrium in temperate Dent and Flint elite lines [21] facilitates the detection of selective sweep signals over sufficiently large genomic regions by the CLR test. On the other hand, the extent of linkage disequilibrium may decrease the power to discriminate between signals caused by genetic hitchhiking due to positive selection and negative background selection in regions with reduced levels of recombination [49, 50]. To assess the number of false-positives due to this effect, we explored the recombination landscape in the Dent and Flint panels by estimating lower bounds of historical recombination events [51]. The proportion of candidate genes located in regions with strongly reduced recombination rates and high linkage disequilibrium like (peri-) centromeric regions was then estimated. We found that 74.8 % of the Dent and 80.9 % of the Flint candidates were not located in regions with low levels of recombination (10 % quantile per chromosome; Additional file 1: Figure S2) indicating that the majority of candidates represent targets of selection rather than false-positive signals. Furthermore, in a classic selective sweep scenario (in contrast to background selection) targets of selection are to be enriched for derived alleles. As an additional test of our candidate regions, we included information from *Sorghum bicolor* to distinguish between ancestral and derived alleles. The Dent and Flint candidate gene sets revealed significantly higher derived allele frequencies compared to the remaining genes as measured by Fay and Wu’s normalized *H* [52] (*p* < 2.2e-16; Additional file 1: Table S4), which also supported positive selection as the driving force of the observed allele frequency changes.

### Gene ontology and pathway analyses of candidate gene sets

Considering genetic differentiation and distinct phenotypic characteristics of Dent and Flint, we tested whether the candidate gene sets were enriched for specific biological processes or pathways. Gene ontology (GO) terms associated with the identified genes were available for around 40 % of the candidates (333 for Dent and 214 for Flint). No significant GO term enrichment of biological processes, cellular components, and molecular functions could be detected for either of the two sets (Additional file 1: Figure S3). To investigate if candidate genes revealed a pool-specific enrichment for metabolic pathways, we performed pathway analyses using MapMan [53]. Based on information available for 58 Dent and 40 Flint candidate genes, we observed a grouping of genes associated with tetrapyrroles (chlorophyll and heme precursors) for Dent and for terpenoid metabolism for Flint (Additional file 1: Figure S4). The latter included the two genes *ZmPPS7.3* (*GRMZM2G014508*) and *ZmPPS8.2* (*GRMZM2G483889*), which encode a large and a small subunit of the geranyl diphosphate synthase complex in maize, respectively [54]. Like their homologues in *A. thaliana* [55], they are assumed to be involved in the biosynthesis of precursors of hormones from the isoprenoid pathway (e.g. gibberellins, brassinosteroids, and abscisic acid). The ability to produce other downstream products of this enzyme, namely *β*-caryophyllenes, has been shown to differ between European Flint and U.S. Dent lines and suggested that this defense response signal against herbivores was largely lost in temperate U.S. Dent [56, 57]. The analysis of candidates associated with other traits that are known to differentiate Dent and Flint revealed six Flint candidates that, according to GO terms, are related to cold tolerance, a trait that is characteristic for temperate Flint [24]. For two of the candidates, differential expression upon exposure to chilling temperature has been reported in maize (*GRMZM2G035584* [58] and *GRMZM2G095562* [59]) as well as for the homologous gene of *GRMZM2G139680* in rice [60]. The molecular and functional characterization of the identified candidate genes in maize and the investigation of differences between Dent and Flint in the regulation of phytohormone pathways or secondary metabolism may provide further insights in the adaptation of maize to different environments. Up to now, comprehensive RNA expression data across various developmental stages and tissues are mainly available for U.S. Dent lines like B73, which underlines the need for a better structural and functional genomic characterization of the Flint germplasm pool and its unique properties.

### Assessing the phenotypic effects of candidate genes on flowering time in a Dent–Flint introgression library

In the genome-wide selection screens, we identified 18 candidates for Dent and 12 candidates for Flint, which could be assigned to the flowering pathway based on previous reports in maize, GO terms, and/or sequence homology to flowering genes characterized in other species [30, 32–34, 61, 62]. We focused exemplarily on candidate genes associated with the flowering network in maize as flowering time is an important agronomic trait that differentiates temperate Dent and Flint. However, functional studies of these genes in maize were available for only 30 % of the candidates (Additional file 4: Table S5). Here, we investigated the effect of the flowering time candidate genes in more detail using a maize introgression library.

**Fig. 2:**
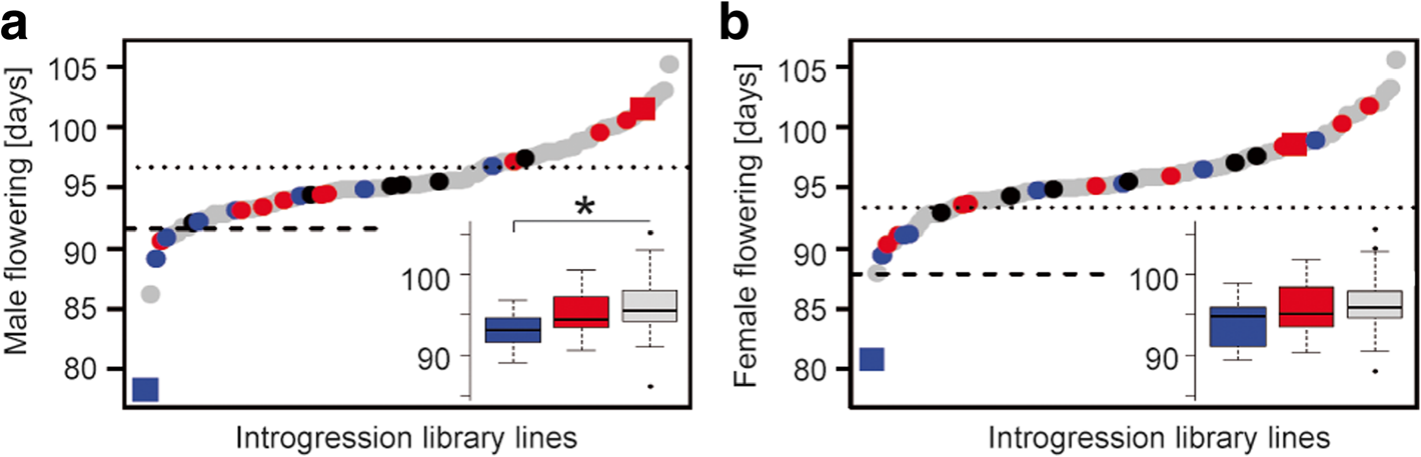
Effect of candidate genes on flowering time in a Dent–Flint introgression library. Adjusted means of (**a**) male and (**b**) female flowering times for 97 introgression lines (*circles*) and the Dent and the Flint parental line (*red and blue squares*, respectively). Lines carrying a segment with Dent or Flint flowering time candidate genes are highlighted in *red* or *blue*, respectively, and lines with a Dent and a Flint candidate are shown in *black*. The *dotted and dashed lines* represent the significance thresholds without (α = 0.05) and with correction for multiple testing (α = 0.05/97). *Boxplots* of adjusted means of flowering times are depicted in the lower parts of (**a**) and (**b**) for seven lines carrying Flint haplotypes of Flint flowering time candidates (*blue*), nine lines carrying Flint haplotypes of Dent flowering time candidates (*red*), and the 75 lines not carrying a flowering time candidate (*gray*). For details about the respective lines see Additional file 5: Table S6. *Boxplots* show the upper and lower quartile, median (*horizontal bar*), and whiskers (*vertical bars*) of the adjusted means. Points above and below the whiskers indicate values ± 1.5 times the interquartile range. Significance of Student’s *t*-tests with *p* < 0.05 is indicated by *

The introgression library had a Dent genetic background with introgressions from a Flint donor line and comprised 97 lines, which carried single Flint segments and covered in total 50.9 % of the Flint donor genome (1048.7 Mb) with a median length of the donor genome segment size of 10.6 Mb (average: 30.8 Mb; Additional file 5: Table S6). We obtained phenotypic data for male and female flowering time based on a field experiment carried out at two locations in Germany. Heritabilities were 0.60 (CI_0.95_ = [0.40; 0.73]) and 0.51 (CI_0.95_ = [0.27; 0.67]) for male and female flowering time, respectively. Phenotypic differences between the Dent and Flint parent were larger for male than for female flowering time (23.2 and 17.8 days, respectively). Based on the least significant difference (α = 0.05), 63 (64.9 %; Fig. 2a) and 16 lines (16.5 %; Fig. 2b) differed significantly from the recurrent Dent parent for male and female flowering time, respectively. Fifteen of these lines had significant effects for both male and female flowering time (α = 0.05). When correcting for multiple testing (α = 0.05/97), six lines (6.2 %) differed significantly for male and none for female flowering time.

Of the 97 lines, 22 carried a Flint introgression harboring one or several of the flowering time candidates identified in the selection screens (Additional file 5: Table S6). Fourteen of the 30 candidates were represented in these 22 introgression lines. Seven lines carried a segment with one or more of seven flowering time candidates identified in Flint and nine lines carried one or more of six flowering time candidates identified in Dent. Six lines carried a segment with a combination of Dent and Flint candidates. Although 75 lines did not carry one of the flowering time candidates identified in our selection screens, they may carry other flowering time genes with alleles differing between the Dent and the Flint parent of the introgression library. Lines carrying the Flint haplotype of a Flint candidate differed significantly from the 75 lines which did not carry one of the flowering time candidates from the selection screens (93.1 versus 96.1 days, *p* value = 0.011; Fig. 2a). For the lines which carried the Flint haplotype of a Dent selection candidate, this difference was not significant. The results indicate that in the genetic material under study, the Flint haplotypes of Flint candidates promoted flowering time more than the Flint haplotypes of Dent candidates.

Of the six lines with significant difference in male flowering compared to the Dent parent after correcting for multiple testing (α = 0.05/97), two carried Flint haplotypes of Flint candidates and one a Flint haplotype of a Dent candidate. One of the lines included the well-characterized large-effect region comprising the ethylene-responsive transcription factor *Rap2* (related to *APETALA2 7*, *ZmRap2.7*, *Rap2*, *GRMZM2G700665*) and its regulatory upstream locus *Vgt1* [62], a major QTL for flowering time in maize [29, 30]. The other line contained *Zcn1* (one of several members of the *ZEA CENTRORADIALIS* or *TERMINAL FLOWER1* (*TFL1)*-like gene family [32]; also *Phosphatidylethanolamine-binding protein1*, *Pebp1*, *GRMZM2G092008*), for which so far only a moderate effect on flowering time was reported in maize [63]. This gene is related to *TFL1* in *A. thaliana* [32], which is an antagonist of the *FLOWERING LOCUS T* (*FT*) [64, 65] and required for the maintenance of an indeterminate inflorescence meristem identity and the regulation of flowering time in *A. thaliana* and maize [63–66]. The line with the Dent candidate carried *Zmm22* (*MADS-transcription factor 69*, *Mads69*, *GRMZM2G171650*), which was recently reported to be associated with variation in flowering time in maize [67] and is considered a candidate for maize domestication and/or improvement [68, 69].

Overall, our findings in the introgression library support the relevance of the investigated genomic regions and their associated candidates for promoting flowering time and confirm the quantitative nature of flowering time in maize, determined by many genes with small effects [26] and only few genes with larger effects. Here, the effects of *Zcn1* and *Zmm22* were stronger than reported previously, which may be attributed to a stronger substitution effect when replacing a Dent haplotype with a Flint haplotype. We will target potential expression differences of flowering time candidates in the Dent–Flint introgression library in future studies to characterize possible differences in the regulation of the flowering network between germplasm pools adapted to different environments.

### Differential selection on components of the flowering network within temperate maize

**Fig. 3:**
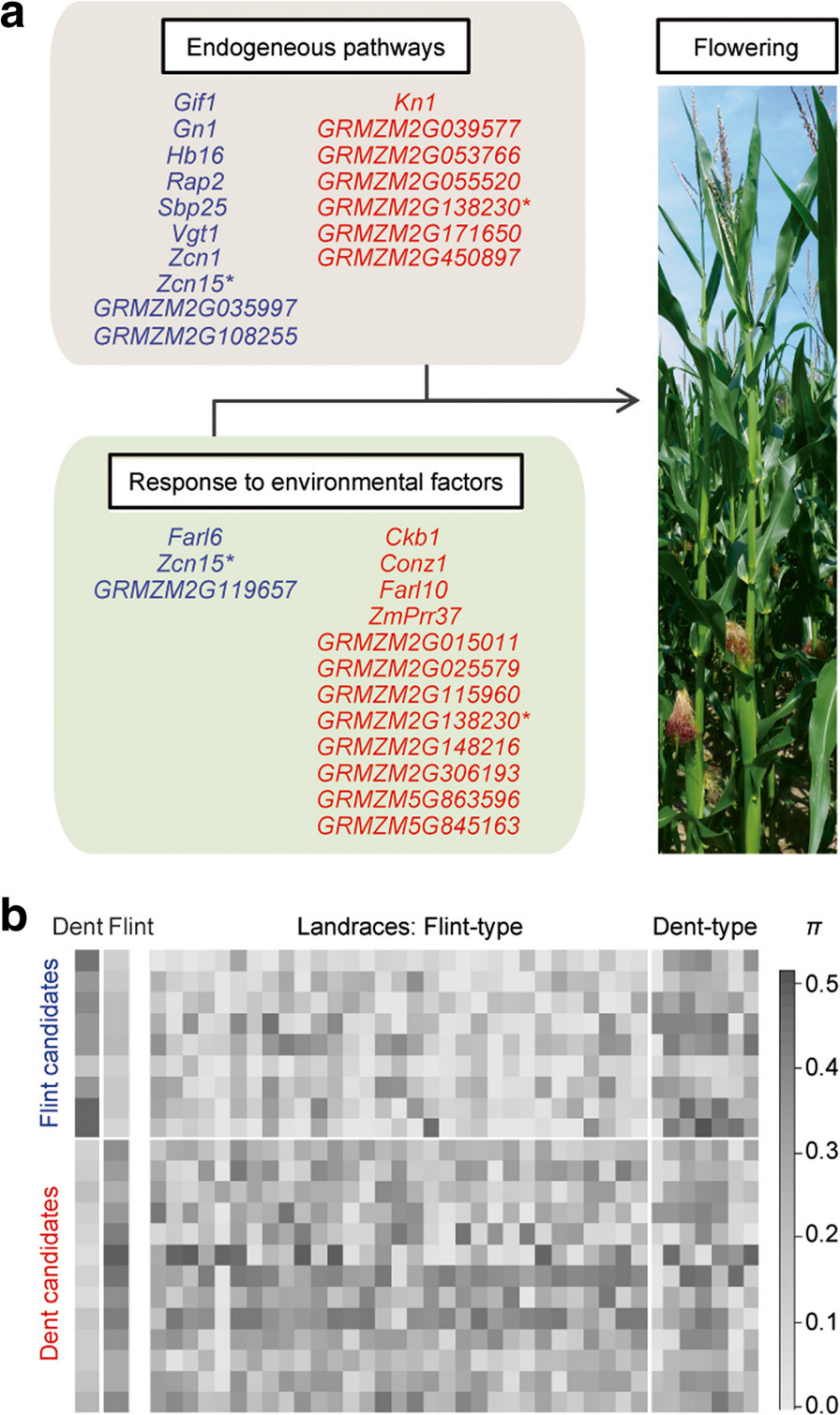
Selection candidates of the maize flowering network and their nucleotide diversity in 136 elite lines and 38 European landraces. **a** Candidates associated with the maize flowering network identified under selective pressure in 70 Dent (*red*) and 66 Flint (*blue*) lines based on genotyping data. Candidates are grouped according to their putative function in endogenous pathways and pathways regulated by environmental factors. For details about the candidate genes and their classification, see Additional file 4: Table S5. Ambiguous assignments according to GO annotations and literature are indicated by *. **b** Nucleotide diversity *π* of nine Flint (*blue*) and 13 Dent (*red*) flowering time candidate genes for 136 temperate elite lines as well as 31 Flint-type and seven Dent-type European landraces. Mean values for each gene were calculated for the panels of Dent and Flint elite lines (*left*) and for each of the 38 landraces (*right*). For details about candidate genes, gene-wise *π* values, and order of landraces, see Additional file 6: Table S8

We investigated the 30 flowering time candidates with respect to their assignment to endogenous pathways and pathways regulated by environmental factors within the flowering network to determine if different components of the flowering network were under selective pressure in Dent and Flint, respectively. Within the flowering network, Flint candidates were involved predominantly in endogenous signaling, hormone-dependent, and developmental processes (10 of 12 candidates, 83.3 %), whereas the Dent candidates indicated a prevalence for response to environmental factors like light and photoperiod (12 of 18 candidates, 66.7 %; Fig. 3a, Additional file 4: Table S5). As described above, Flint candidates included the well-characterized *Rap2*/*Vgt1* locus and *Zcn1*. Furthermore, we found the *Squamosa promoter binding protein-transcription factor 25* (*Sbp25*, *GRMZM2G414805*) and *Gnarley1* (*Gn1*; also *Homeobox protein KNOTTED1-like 4*, *Knox4*, *GRMZM2G452178*) that are associated with aging and hormone-dependent pathways (Additional file 4: Table S5). *Gn1* is likely to act upstream of the “green revolution” gene encoding gibberellin 20-oxidase [70] and to regulate *Gibberellin 2-oxidase 1* expression in maize, thus influencing vegetative to reproductive phase transition, pollen tube growth, and stem elongation by changing the availability of gibberellin [71]. *Gibberellin 2-oxidase 1* is additionally regulated by *Knotted1* (*Kn1*, *GRMZM2G017087*) which was identified as a Dent candidate gene [71]. Another well-characterized Dent candidate is *Constans1* (*Conz1*, *GRMZM2G405368*), which is a putative ortholog of the photoperiod genes *CONSTANS* from *A. thaliana* and *Heading date1* in rice [72]. To the best of our knowledge, 20 of the 30 detected flowering time candidates have not yet been functionally characterized in the context of maize flowering time, but were associated with the flowering network based on GO terms or reports in other species such as *A. thaliana* and rice (Additional file 4: Table S5). Thus, our study revealed candidates that warrant further investigation of their functional relevance in maize flowering time. Based on the observed allele frequency differences of the candidate genes within the 136 elite lines and with respect to their function in maize or, for example, *A. thaliana*, we hypothesize that different components of the flowering network were under selective pressure in Dent and Flint. The Flint-specific haplotypes of these genes might constitute a promising source for the adaptation of maize germplasm pools to shorter vegetation periods.

### Diversity of flowering time candidates in elite lines and European landraces

**Fig. 4:**
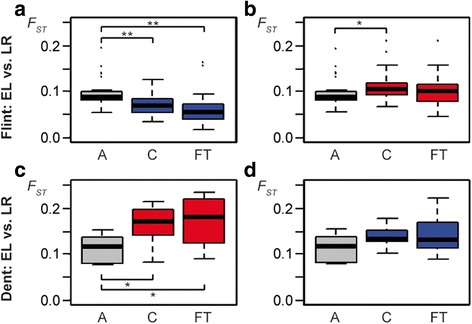
Differentiation between elite lines (EL) and landraces (LR) for candidate genes. The upper panel shows the differentiation (*F*_*ST*_) between 66 Flint elite lines and 31 Flint-type landraces for (**a**) Flint (*blue*) and (**b**) Dent (*red*) candidate gene sets. The lower panel depicts the differentiation between 70 Dent elite lines and seven Dent-type landraces for (**c**) Dent (*red*) and (**d**) Flint (*blue*) candidate gene sets. The boxplots show *F*_*ST*_ values for all (A; *gray*) genes except the candidates, the candidate (C) genes, and for the subset of candidates associated with flowering time (FT). *Boxplots* show the upper and lower quartile, median (*horizontal bar*), and whiskers (*vertical bars*) of the *F*_*ST*_ values. Points above and below the whiskers indicate values ± 1.5 times the interquartile range. Significance of two-sided Wilcoxon rank sum tests with *p* < 0.05 are indicated by * and with *p* < 0.001 by **. For details see Additional file 7: Table S9

As most of the European Flint inbred lines are assumed to be derived from few landraces [44], we compared the diversity and the allelic composition of 22 flowering time candidates (13 Dent and 9 Flint candidates tagged by at least five SNPs) between the elite lines and a unique panel of 38 European landraces (Additional file 1: Table S7). For each landrace, 22 to 24 plants were genotyped at high density with the Axiom® Maize Genotyping Array [21]. The majority of the landraces (N = 31) had Flint-type kernels. These landraces exhibited lower levels of diversity in the Flint flowering time candidates (gene-wise average: *π* = 0.130) compared to the Dent flowering time candidates (*π* = 0.243), thus confirming the pattern found in the Flint elite lines (Fig. 3b, Additional file 6: Table S8). We further investigated the level of differentiation between Flint-type landraces and Flint elite lines and observed low levels of *F*_*ST*_ for the Flint flowering time candidates (*F*_*ST*_ = 0.060; Fig. 4a, FT) with ten landraces from France, Germany, and Spain displaying values even smaller than 0.050 (Additional file 7: Table S9). These low values of differentiation suggested a major contribution of the Flint-type landraces to the flowering time candidate gene diversity observed in the Flint elite lines. This hypothesis was corroborated by the finding that the entire set of Flint candidate genes also revealed significantly lower levels of differentiation compared to all other genes, which were not under differential selection between Dent and Flint elite lines (*F*_*ST*_ = 0.072 versus 0.095, *p* value = 6.0e-04; Fig. 4a, A versus C).

Consistent with the hypothesis that Flint elite lines and Flint-type landraces have a common history, significantly higher levels of differentiation were observed for Dent candidate genes compared to all remaining genes (*F*_*ST*_ = 0.111 versus 0.095, *p* value = 0.017; Fig. 4b, A versus C). Together, these findings indicated that the reduced diversity observed for Flint candidate genes in Flint elite lines was already present in a broad panel of European landraces and that the candidate gene diversity of the Flint elite lines originate from a limited number of Flint-type landraces used for elite line development in some historically important breeding centers [44].

The remaining seven landraces displayed at least partially Dent-type kernels. These landraces revealed high levels of diversity for Dent and Flint flowering time candidates (*π* = 0.225 and 0.260, respectively; Fig. 3b, Additional file 6: Table S8) and showed a high level of differentiation with Dent elite lines for the Dent flowering time candidates (*F*_*ST*_ = 0.170; Fig. 4c, FT). The same pattern was found in the analysis of the entire Dent candidate gene set, which revealed significantly higher levels of differentiation compared to all remaining genes (*F*_*ST*_ = 0.164 versus 0.111, *p* value = 0.026; Fig. 4c, A versus C), but no significant difference for Flint candidates compared to all remaining genes (*F*_*ST*_ = 0.138 versus 0.112, *p* value = 0.209; Fig. 4d, A versus C; Additional file 7: Table S9). These results indicated that the European Dent-type landraces exhibit a different allelic composition in the Dent candidates compared to the Dent elite lines and did most likely not contribute to the Dent elite material under study.

### Selection on upstream and genic regions of the candidates

To examine how specific elements of the genic regions contributed to the differentiation between Dent and Flint, we compared levels of differentiation for 5 kb and 500 bp upstream regions, genic regions, and exons between the candidate gene sets and all remaining genes. To increase the resolution of our analyses, we investigated the candidate gene sets based on whole-genome sequence data of 40 temperate elite lines (21 Dent and 19 Flint) [21, 22], which were part of the panel of 136 elite lines genotyped with the 600 k array with the exception of three lines (Additional file 1: Table S1). Based on 13,246,294 bi-allelic SNPs, we observed a significant reduction of mean *π* and TD in 727 Dent and 403 Flint candidate genes tagged by at least five SNPs (of in total 876 and 545 candidates, respectively) compared to 31,163 remaining genes (*p* value < 2.2e-16; Additional file 1: Figure S5 and Additional file 1: Table S4). *F*_*ST*_ values calculated between Dent and Flint were significantly higher for candidate gene sets compared to all remaining genes for 5 kb and 500 bp upstream as well as genic and exonic regions. Together, these findings supported the results obtained from the selection screens in the panel of 136 temperate inbred lines genotyped with the 600 k array.

Previous studies in maize suggested an important role of the divergence of regulatory elements in the context of domestication [73–75]. In our study, distributions of *F*_*ST*_ values were comparable for 5 kb and 500 bp upstream as well as genic and exonic regions in each of the two candidate gene sets (Additional file 1: Figure S6 and Additional file 1: Table S4). However, the power to resolve whether selection acted differentially in upstream and genic regions was probably limited by the high level of linkage disequilibrium observed in temperate Dent and Flint lines [21]. The outcome of ongoing large-scale whole genome and transcriptome sequencing will allow the investigation of the impact of selection on the regulation of gene activity in the two pools and its consequence for the genomic differentiation between Dent and Flint.

## Conclusions

In this study, we report genomic differentiation between two major temperate maize germplasm pools, Dent and Flint. By comparing a representative panel of Dent and Flint elite lines, we identified candidate genes under differential selective pressure in Dent and Flint. The significant enrichment in derived allele frequencies for these genes provided strong indication that the candidate regions represented selective sweeps. Candidate genes associated with agronomic traits known to differ between Dent and Flint could be identified. Most of the detected flowering time candidates have not yet been functionally characterized in maize. Investigating the effect of the flowering time candidates in a Dent–Flint introgression library, we found that Flint haplotypes of these candidates promoted earlier flowering. Within the flowering network of maize, a Flint-specific enrichment of genes associated with endogenous signaling, hormone-dependent pathways, and developmental processes was discovered in contrast to Dent, where selection seemed to act predominantly on genes involved in the response to environmental factors. Low levels of differentiation of Flint flowering time candidate genes between European Flint elite lines and European landraces indicated that the allelic composition of the elite lines was comparable to those of the Flint-type landraces and suggested a major contribution of landraces from France, Germany, and Spain to the candidate gene diversity in the Flint elite lines. Our findings highlight the role of genomic regions that have undergone intense selection and contributed to the differentiation of temperate Dent and Flint with likely effects on different agronomic traits. The identification of pool-specific selection signatures enabled insights into different patterns of diversity between temperate Dent and Flint and provides new targets for future functional analyses and crop improvement.

## Methods

### Plant material and genotyping of elite lines and landraces

The 136 elite inbred lines (Additional file 1: Table S1) were selected to represent the genetic diversity of European and American temperate maize and were genotyped with the 600 k Affymetrix® Axiom® Maize Array described in [21]. Landraces were selected to reflect the genetic and phenotypic diversity within Central and Western Europe (Additional file 1: Table S7) and were represented by 22 to 24 plants each. All 906 landrace individuals were genotyped using the 600 k array and the genotype cluster model file (http://www.affymetrix.com/catalog/prod820010/AFFY/Axiom%26%23174%3B-Maize-Genotyping-Array).

If not denoted otherwise, analyses were performed using R version 3.0.1 [76]. SNP positions were assigned to the reference sequence B73 v2 [22] for all datasets. Analyses of the elite line panel were based on 566,961 best quality SNPs [21] with heterozygous calls masked as missing. Indels, unmapped markers, SNPs with ≥ 10 % missing values in the elite line panel [21], markers designed to specifically differentiate between two Dent lines [77], and monomorphic SNPs were excluded, resulting in 547,412 SNPs for analyses of temperate elite lines. Analyses including the landraces were based on a subset of 486,208 SNPs.

### Population structure analysis and estimation of historical recombination rates

For population structure analyses of the genotyped panel of 136 elite lines, missing data were imputed using Beagle [78] version 3.3.1 via the R package “synbreed” [79] version 0.10-3. A linkage disequilibrium pruning step was performed applying an *r*^*2*^ threshold of 0.8 followed by the estimation of ancestry using ADMIXTURE [80] version 1.23. To obtain an estimate of the historical recombination rates in Dent and Flint based on the genotype data of the elite line panel, the four-gamete test [51] was calculated. This test gives a conservative estimate (i.e. the minimum number) of recombination events in the history of a sample. Values for the pairwise tests of neighboring SNPs were averaged over 1000 sites and the mean of each 1000 site bin was plotted. Reported were recombination events per Mb and regions of low recombination rates were defined as regions exhibiting rates within the 10 % quantile per chromosome.

### Screens for selection signatures

Nucleotide diversity *π* [9] and Tajima’s *D* (TD) [10] were calculated for each panel of inbred lines using a customized script. The fixation index *F*_*ST*_ [45] was calculated across the two panels using PLINK [81] version v1.90b2m. Metrics were calculated per SNP and averaged over windows of 40 SNPs (sliding by 10 % and corresponding to an average physical distance of 10 kb), using the R package “zoo” [82]. The grid-based composite likelihood ratio (CLR) test was calculated for each panel as implemented in SweepFinder [11]. For CLR, the size of the non-overlapping grids was 150 kb, which is the same magnitude as the maximal distance between two SNPs with *r*^*2*^ > 0.2 in the elite line panel [21]. Windows exhibiting values within the 10 % quantile (*π*, TD) and the 90 % quantile (*F*_*ST*_, CLR) were submitted for candidate gene analysis based on the B73 v2 [22] annotation, version 5b60 (ftp://ftp.gramene.org/pub/gramene/maizesequence.org/release-5b/filtered-set/), containing 39,656 gene models. GO terms were tested for enrichment using the resources developed by [83] based on maize gene IDs by applying a hypergeometric test with a Benjamini–Yekutieli correction ([84]; FDR = 0.05) to account for multiple tests. Pathway analysis was performed using MapMan version 3.5.1 [53] based on the mapping of the first transcript of each gene to the file Zm_B73_5b_FGS_cds_2012.m02 downloaded from the MapMan webpage (http://mapman.gabipd.org/web/guest/mapmanstore). For visualization, arbitrary values of –4.5 and 4.5 were assigned to Dent and Flint candidate gene transcripts, respectively.

### Whole-genome sequence datasets

For Fay and Wu’s normalized *H* [52], the maize-sorghum genome alignment (http://pipeline.lbl.gov/downloads.shtml) was parsed with a custom Perl script to obtain the nucleotide in sorghum representing the ancestral maize allele for 298,388 SNPs of the genotyped panel of elite lines. Whole-genome sequence data for 30 elite lines were used to call SNPs and small indels by employing an integrative analysis pipeline [21] and filtering for high mapping (MQ ≥ 30) and genotyping quality (GQ > 5), major allele frequency ≥ 90 %, a minimal distance of 3 bp between adjacent SNPs, and a minimal and maximal coverage by three and 60 MQ30 reads for lines sequenced to medium coverage, respectively. The latter criterion was adjusted for four deep sequenced lines requiring ten and 300 MQ30 reads, respectively. We combined the obtained marker set with whole-genome sequence data from ten temperate inbred Dent and Flint lines from the maize HapMap2 project [17, 22] resulting in a VCF file including 13,246,294 bi-allelic SNPs. We filtered for SNPs with ≤ 50 % missing values across the 40 lines (*F*_*ST*_) and within germplasm pools (*π* and TD). The combined VCF file was converted to hapmap format with a customized Perl script, and *π* and TD per gene were obtained with Variscan version 2 [85] with runmode “12”.

For gene-wise calculations based on the genotyped panels of 136 elite lines and 38 European landraces (normalized *H*, *F*_*ST*_) or on whole-genome sequence data of 40 elite lines (*π* and TD), the genic region of the longest protein-coding transcript, including 5 kb upstream, was used and metrics were calculated if at least 5 SNPs were available for analysis. For a separate analysis of exonic, genic, 500 bp, and 5 kb upstream regions, *F*_*ST*_ was determined in case of at least 5 SNPs per region based on whole-genome sequence data using vcftools v0.1.11 [86]. Two-sided Wilcoxon rank sum tests [87] were performed on gene-wise metrics to test for differences between candidate genes and remaining genes within pools.

### Introgression library

To represent the Flint genome as introgression segments in a Dent genetic background, a European Dent inbred line was crossed with a European Flint inbred line, followed by backcrossing, marker-assisted selection, and several rounds of selfing [88]. The Dent parental line originated from south-eastern Europe and exhibits a high general combining ability for kernel yield. The Flint parental line was selected for high general combining ability for biomass yield and good performance in Central European climates. The introgression library used in the present study comprised 535 lines, with 97 lines carrying a single segment of the Flint parent. To estimate the length of individual donor genome fragments, the distance between markers on the respective donor genome fragment plus half the distance to the adjacent marker flanking the donor genome fragment on either side of the fragment was calculated. For the introgression lines, the two parental lines, and a check, male and female flowering times were obtained from two field experiments conducted in 2014 at the German trial locations Roggenstein (N 48°11′13.24″, E 11°19′50.86″, 517 m AMSL, average temperature in 2014: 9.8 °C) and Freising (N 48°24′11.62″, E 11°43′21.99″, 480 m AMSL, average temperature in 2014: 9.7 °C). Each experiment was laid out as an α–lattice design with two replications, except for parental lines and the check that were repeated three and five times, respectively. Male and female flowering time was recorded as days after sowing until 50 % of plants per plot exhibited emerged anthers and silks, respectively. Adjusted means for flowering time were calculated using Plabstat [89]. The difference in adjusted means between the 97 single-segment introgression lines and the Dent parental line was tested at a significance level of α = 0.05 and α = 0.05/97 to correct for multiple testing. Adjusted means of flowering times for seven lines carrying Flint haplotypes of Flint flowering time candidates, nine lines carrying Flint haplotypes of Dent flowering time candidates, and the 75 lines not carrying a flowering time candidate gene were tested for significant differences by calculating Student’s *t*-test.

## Additional files

Additional file 1: Figure S2. Variation of historical recombination rates along the ten maize chromosomes. **Figure S3.** Gene ontology (GO) terms assigned to categories of biological processes for the candidate gene sets. **Figure S4.** Pathway analysis for the Dent and Flint candidate gene sets using MapMan [53]. **Figure S5.** Gene-wise *π* (*left*) and TD (*right*) values based on whole-genome sequence data of 21 temperate Dent and 19 temperate Flint lines. **Figure S6.** Distributions of *F*_*ST*_ values for different genomic regions based on whole-genome sequence data of 40 elite lines. **Table S1.** Elite lines under study with germplasm pool assignment and data source. **Table S2.** Window-based statistics and thresholds according to the selected quantile based on the panel of 136 temperate inbred lines genotyped with the 600 k array. **Table S4.** Gene-wise statistics for all genes and for Dent and Flint candidate genes. **Table S7.** Landraces under study with their geographic origins. (PDF 1175 kb) Additional file 2: Figure S1. Metrics of the selection screens for 136 temperate inbred lines along the ten maize chromosomes based on genotyping data. (PDF 4242 kb) Additional file 3: Table S3. Gene-wise statistics for 1407 candidate genes identified in the 70 Dent (N = 876) and 66 Flint (N = 545) lines based on genotyping data. (XLSX 1238 kb) Additional file 4: Table S5. Pathway assignment and information about candidates associated with the flowering network in maize. (XLSX 50 kb) Additional file 5: Table S6. Genomic composition and flowering dates of 97 single-segment introgression library lines. (XLSX 114 kb) Additional file 6: Table S8. Gene-wise nucleotide diversity *π* and *F*_*ST*_ for flowering time candidate genes with at least 5 SNPs in the genic and 5 kb upstream region. (XLSX 24 kb) Additional file 7: Table S9. Gene-wise level of differentiation of allele frequencies between landraces and Dent (left) and Flint (right) elite lines based on genotyping data. (XLSX 27 kb)
