# Supplementary material for: A comprehensive study of the genomic differentiation between temperate Dent and Flint maize
Source: Genome Biol. 2016 Jul 8;17:137. doi: 10.1186/s13059-016-1009-x (PMC4937532; doi:10.1186/s13059-016-1009-x)
Supplement: Additional file 1: Figure S2. — Variation of historical recombination rates along the ten maize chromosomes. Figure S3. Gene ontology (GO) terms assigned to categories of biological processes for the candidate gene sets. Figure S4. Pathway analysis for the Dent and Flint candidate gene sets using MapMan [53]. Figure S5. Gene-wise π (left) and TD (right) values based on whole-genome sequence data of 21 temperate Dent and 19 temperate Flint lines. Figure S6. Distributions of F ST values for different genomic regions based on whole-genome sequence data of 40 elite lines. Table S1. Elite lines under study with germplasm pool assignment and data source. Table S2. Window-based statistics and thresholds according to the selected quantile based on the panel of 136 temperate inbred lines genotyped with the 600 k array. Table S4. Gene-wise statistics for all genes and for Dent and Flint candidate genes. Table S7. Landraces under study with their geographic origins. (PDF 1175 kb) [file 13059_2016_1009_MOESM1_ESM.pdf]

Supplementary Figures

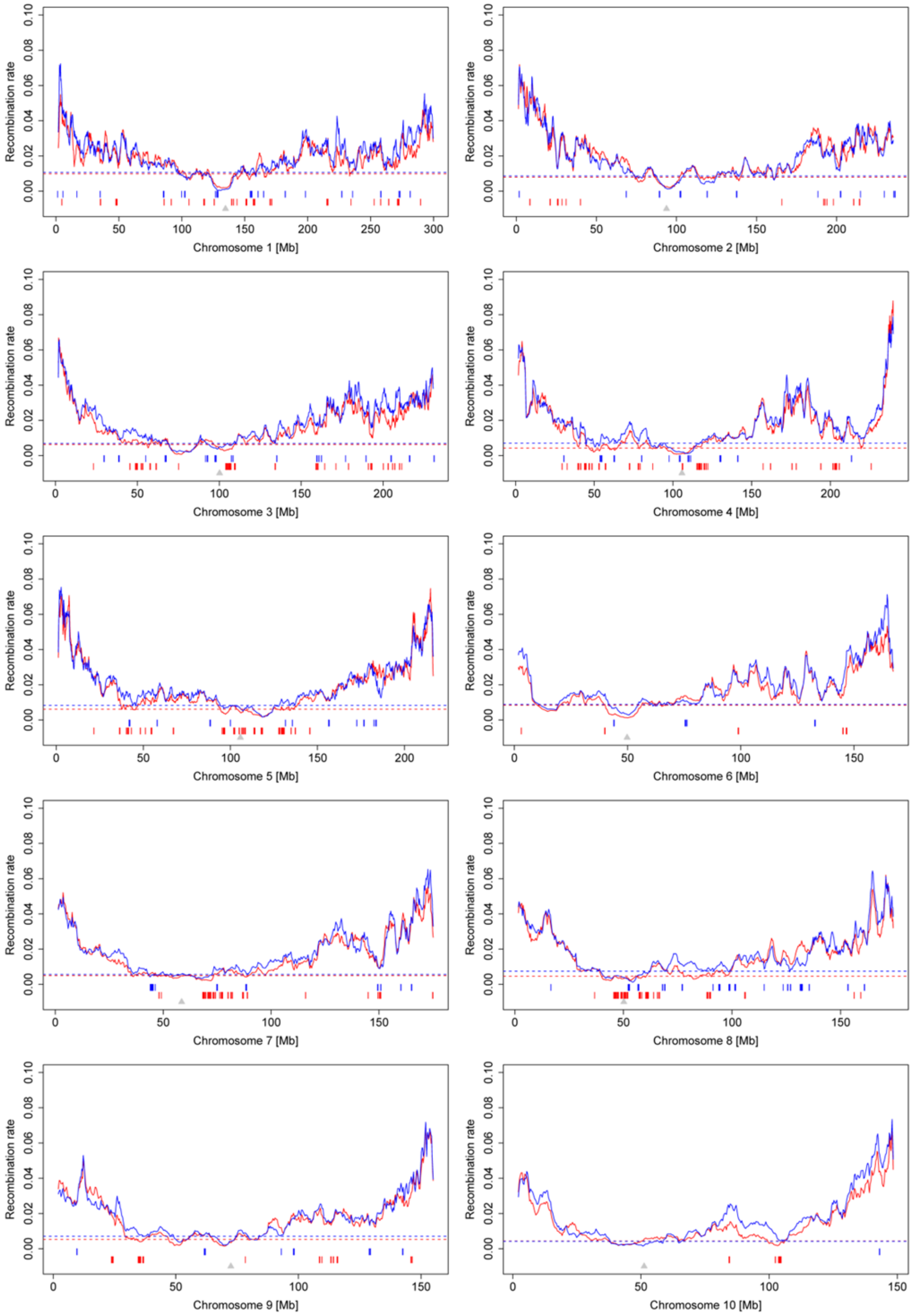

Figure S2: Variation of historical recombination rates along the ten maize chromosomes.
Minimal number of recombination events observed per Megabase (Mb) in 70 Dent (red) and 66 Flint lines (blue) based on genotyping data. The position of candidate genes is highlighted in the corresponding colors; dashed lines indicate the 10% quantile of recombination events per Mb for Dent (red) and Flint (blue) and grey triangles indicate the position of centromeres.

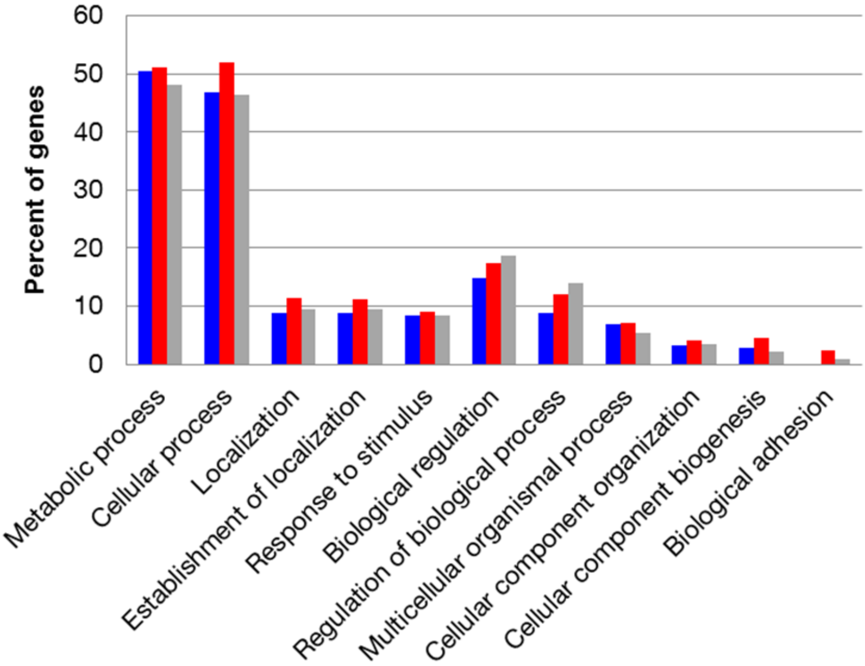

Figure S3: Gene ontology (GO) terms assigned to categories of biological processes for the candidate gene sets. GO terms were available for 333 Dent candidates (red) and 214 Flint candidate genes (blue) compared to the background (N = 25,288; grey).

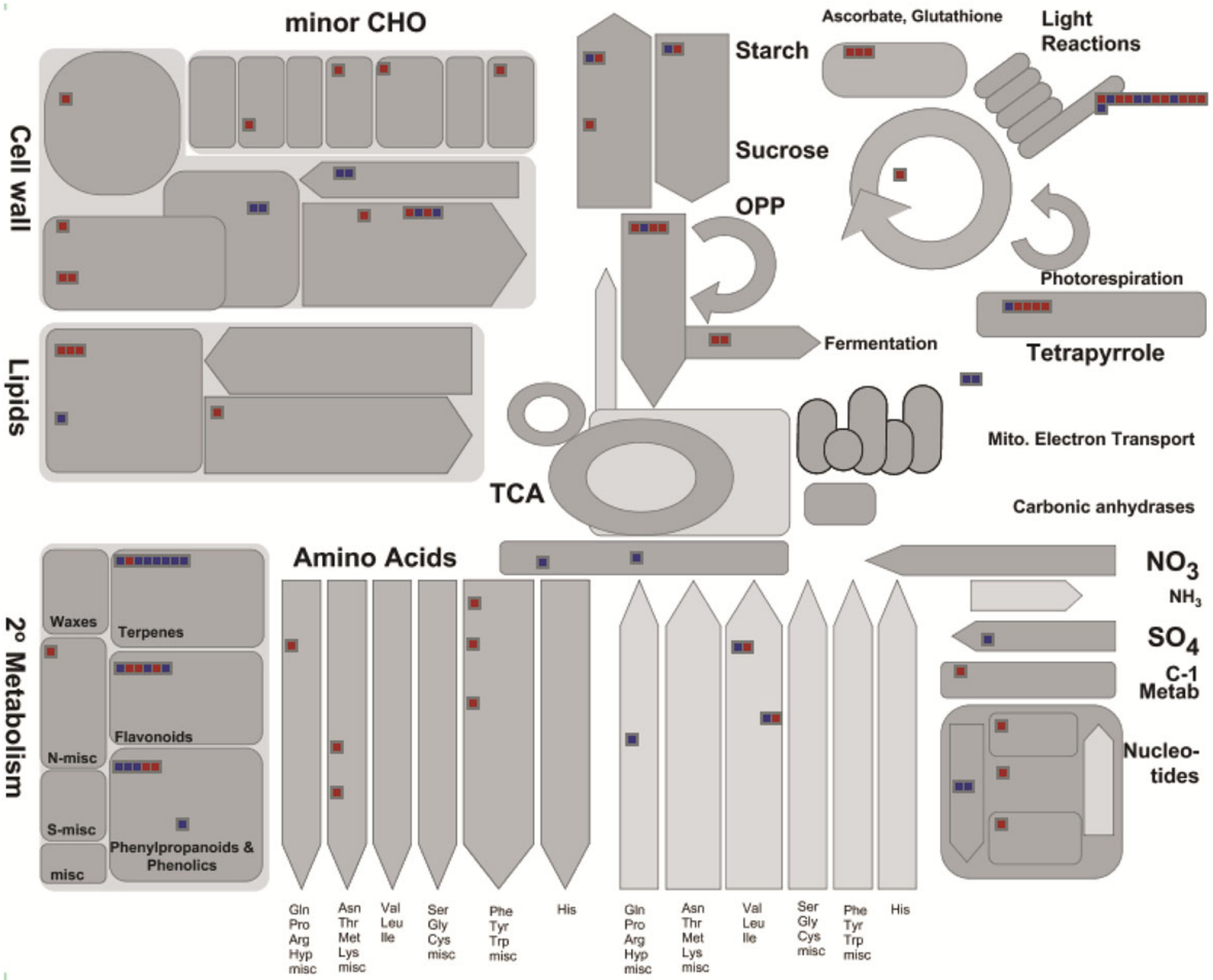

Figure S4: Pathway analysis for the Dent and Flint candidate gene sets using MapMan [53]. Pathway assignments were available for 58 genes for Dent (red squares) and 40 candidate genes for Flint (blue squares) based on the first transcript per gene. For visualization, values of -4.5 and 4.5 were assigned to Dent and Flint candidate gene transcripts, respectively.

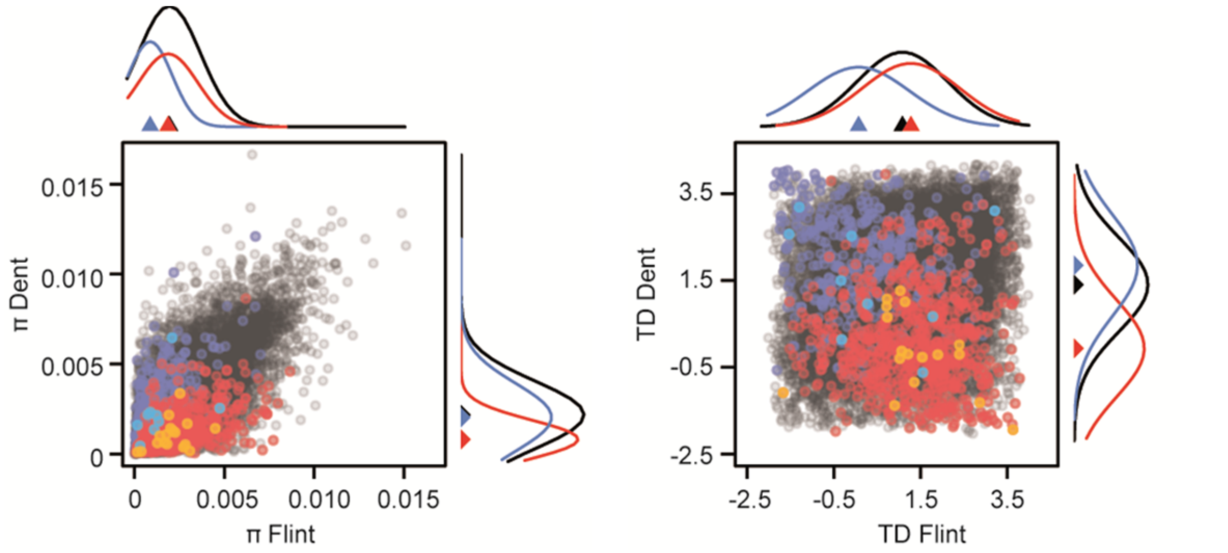

Figure S5: Gene-wise π (left) and TD (right) values based on whole-genome sequence data of 21 temperate Dent and 19 temperate Flint lines. Values for all genes are shown in black, selection candidates are highlighted in red (Dent) and blue (Flint). Candidates associated with the flowering network are labelled in orange (Dent) and light blue (Flint). The relative distribution of values is shown by density plots with means indicated by triangles of the corresponding colour.

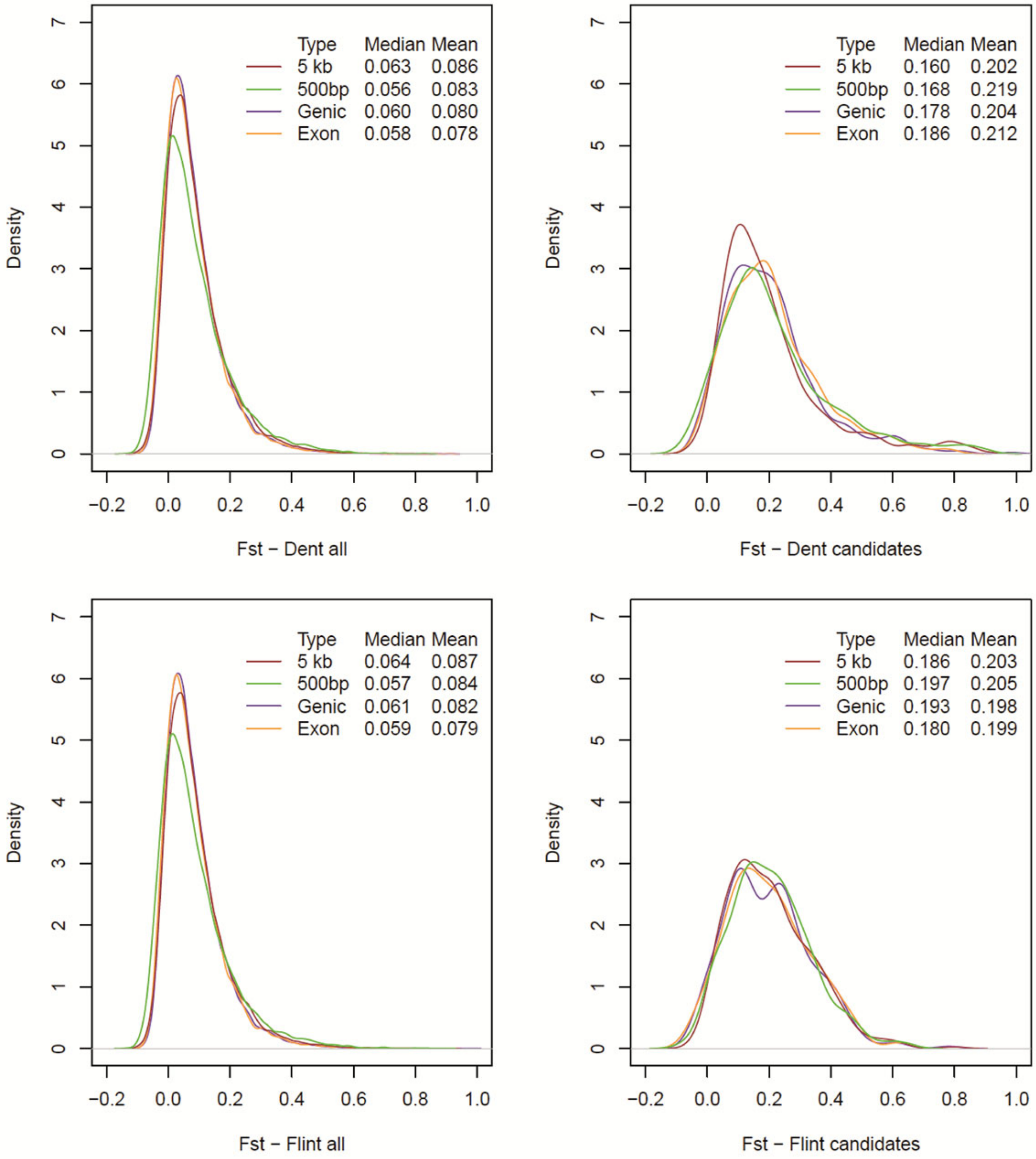

Figure S6: Distributions of F_{ST} values for different genomic regions based on whole-genome sequence data of 40 elite lines. Distributions for 5 kb and 500 bp upstream, genic and exonic regions are shown for all genes except candidate genes on the left and for candidate genes on the right. F_{ST} values in 21 Dent lines are shown in the upper part and values in 19 Flint lines in the lower part. Details are given in **Table S4**.

Supplementary Tables

Table S1: Elite lines under study with germplasm pool assignment and data source.

Pool	Name	Genotyped with the 600 k array	Whole-genome sequence data
Dent	A632	Yes	
	B14	Yes	
	B37	Yes	
	B73	Yes	Unterseer et al.[21]
	B97	Yes	Chia et al.[22]
	CM105	Yes	
	CO125	Yes	
	CR1Ht	Yes	
	D06	Yes	Unterseer et al.[21]
	D09	Yes	Unterseer et al.[21]
	DJ7	Yes	
	EC169	Yes	Unterseer et al.[21]
	F252	Yes	Unterseer et al.[21]
	F288	Yes	
	F353	Yes	Unterseer et al.[21]
	F618	Yes	Unterseer et al.[21]
	F748	Yes	
	F838	Yes	
	F922	Yes	
	F98902	Yes	Unterseer et al.[21]
	Fv113	Yes	
	Fv230	Yes	
	Fv310	Yes	
	K55	Yes	
	Ky21	Yes	Chia et al.[22]
	LH119	Yes	
	LH132	Yes	
	LH149	Yes	
	LH160	Yes	
	LH202	Yes	
	LH38	Yes	
	LH39	Yes	
	LH51	Yes	
	LH54	Yes	
	LH59	Yes	
	LH61	Yes	
	LH65	Yes	
	LH74	Yes	
	LH93	Yes	
	M162W	Yes	Chia et al.[22]
	M37W	Yes	
	MBS847	Yes	
	ML606	Yes	
	Mo17	Yes	Unterseer et al.[21]
	Mo18W	Yes	
	Ms71	Yes	Chia et al.[22]
	Oh43	Yes	Chia et al.[22]
	Oh7B	Yes	Chia et al.[22]
	PA91	Yes	
	PH207	Yes	Unterseer et al.[21]

	PHG29	Yes	
	PHG50	Yes	
	PHG72	Yes	
	PHH93	Yes	
	PHJ90	Yes	
	PHK29	Yes	
	PHK42	Yes	
	PHN82	Yes	
	PHP02	Yes	
	PHR25	Yes	
	PHW17	Yes	
	PHW52	Yes	
	UH250	Yes	Unterseer et al.[21]
	UH304	Yes	Unterseer et al.[21]
	VA85	Yes	
	W117	Yes	Unterseer et al.[21]
	W22	Yes	Chia et al.[22]
	W401	Yes	
	W64A	No	Chia et al.[22]
	WF9	Yes	
	X740	Yes	
Flint	CH10	Yes	Unterseer et al.[21]
	CO255	Yes	
	D102	Yes	
	D118	Yes	
	D141	Yes	
	D142	Yes	
	D143	Yes	
	D152	Yes	Unterseer et al.[21]
	D171	Yes	
	D503	Yes	
	DK105	Yes	Unterseer et al.[21]
	EC49A	Yes	Unterseer et al.[21]
	EM1349	Yes	
	EP1	Yes	Unterseer et al.[21]
	EP42	Yes	
	EP44	Yes	Unterseer et al.[21]
	EZ5	No	Unterseer et al.[21]
	F03802	Yes	Unterseer et al.[21]
	F2	Yes	Unterseer et al.[21]
	F283	Yes	Unterseer et al.[21]
	F4	Yes	
	F41	Yes	
	F471	Yes	
	F48	Yes	
	F564	Yes	
	F64	No	Unterseer et al.[21]
	F7	Yes	Unterseer et al.[21]
	F7012	Yes	
	F902	Yes	
	FC13	Yes	
	FC1571	Yes	
	FC23	Yes	
	FC24	Yes	
	FC25	Yes	
	FC26	Yes	
	FF0721H-7	Yes	Unterseer et al.[21]

FV1	Yes	
FV10	Yes	
FV11	Yes	
FV131	Yes	
FV226	Yes	
FV268	Yes	
FV286	Yes	
FV324	Yes	
FV65	Yes	
FV66	Yes	
FV69A	Yes	
FV70	Yes	
FV79	Yes	
FV83	Yes	
FV85	Yes	
Il14H	Yes	Chia et al.[22]
Lo11	Yes	Unterseer et al.[21]
Lo32	Yes	
P39	Yes	Chia et al.[22]
UH006	Yes	Unterseer et al.[21]
UH007	Yes	Unterseer et al.[21]
UH009	Yes	Unterseer et al.[21]
X1G0895.DH101	Yes	
X1G0895.DH104	Yes	
X1G0895.DH109	Yes	
X1G0896.DH102	Yes	
X1G0896.DH116	Yes	
X1G0896.DH123	Yes	
X1G0896.DH212	Yes	
X1G0897.DH102	Yes	
X1G0897.DH135	Yes	
X1G0897.DH203	Yes	

Table S2: Window-based statistics and thresholds according to the selected quantile based on the panel of 136 temperate inbred lines genotyped with the 600 k array.

Statistic	Pool	Sample size	Min.	Median	Mean	Max.	Threshold
Nucleotide diversity π	Dent	70	0.000	0.318	0.308	0.505	0.213
	Flint	66	0.009	0.318	0.310	0.507	0.220
Tajima´s D	Dent	70	-1.070	0.780	0.730	1.775	0.202
	Flint	66	-1.070	0.723	0.682	1.769	0.169
Composite likelihood ratio	Dent	70	0.000	0.596	4.062	474.000	6.790
	Flint	66	0.000	0.526	6.106	598.100	6.498
Fixation index F_{ST}	Dent vs. Flint	136	-0.014	0.125	0.144	0.804	0.261

Table S4: Gene-wise statistics for all genes and for Dent and Flint candidate genes. Number of genes, mean and median F_{ST} values (mean ± standard error; se) are given. All differences between candidate genes and remaining genes were significant with $p < 2.2e-16$ determined by two-sided Wilcoxon rank sum tests. Data set G refers to 600 k genotyping data and data set S to whole-genome sequence data.

**Statistic**	**Data set**	**Pool**	**All genes**			**Candidate genes**		
			**Number**	**Mean ± se**	**Median**	**Number**	**Mean ± se**	**Median**
Normalized *H*	G	Dent	16,298	-0.198 ± 0.003	-0.127	278	-0.791 ± 0.030	-0.761
	G	Flint	16,421	-0.201 ± 0.003	-0.135	166	-0.649 ± 0.034	-0.659
Nucleotide div. π	S	Dent	32,561	0.002 ± 1.0e-05	0.002	727	0.001 ± 3.9e-05	8.6e-04
	S	Flint	31,971	0.002 ± 9.6e-06	0.002	403	0.001 ± 5.9e-05	8.9e-04
Tajima´s *D*	S	Dent	32,561	1.525 ± 0.006	1.640	727	0.091 ± 0.041	-0.072
	S	Flint	31,971	1.151 ± 0.006	1.225	403	0.168 ± 0.056	0.119
F_{ST} - 5kb upstream	S	Dent	28,563	0.086 ± 5.7e-04	0.063	712	0.202 ± 0.006	0.160
	S	Flint	28,898	0.087 ± 5.8e-04	0.064	377	0.203 ± 0.007	0.186
F_{ST} - 500bp upstream	S	Dent	11,257	0.083 ± 0.001	0.056	282	0.219 ± 0.011	0.168
	S	Flint	11,396	0.084 ± 0.001	0.057	143	0.205 ± 0.010	0.197
F_{ST} - genic regions	S	Dent	22,230	0.080 ± 6.0e-04	0.060	526	0.204 ± 0.007	0.178
	S	Flint	22,469	0.082 ± 6.1e-04	0.061	287	0.198 ± 0.008	0.193
F_{ST} - exonic regions	S	Dent	21,764	0.078 ± 6.1e-04	0.058	400	0.212 ± 0.008	0.186
	S	Flint	21,958	0.079 ± 6.2e-04	0.059	206	0.199 ± 0.009	0.180

Table S7: Landraces under study with their geographic origins.

Name	Abb.[a]	Geographic origin[b]	Source[c]	Plants
Altreier	AL	Altrei, South Tyrol, IT	1	24
Andoain	AN	Andoain, Basque Country, ES	2	24
Barisis	BA	Barisis, Nord-Pas-de-Calais-Picardie, FR	3	24
Bugard	BU	Bugard, Languedoc-Roussillon-Midi-Pyrénées, FR	4	22
Castellote	CA	Castellote, Aragon, ES	2	24
Colmar	CO	Colmar, Alsace-Champagne-Ardenne-Lorraine, FR	3	24
Fleimstal	FL	Fiemme Valley, South Tyrol, IT	1	24
Gazost	GA	Gazost, Languedoc-Roussillon-Midi-Pyrénées, FR	3	24
Gelber Badischer Landmais	GB	Upper Rhine valley, DE	4	24
Gleisdorfer	GL	Gleisdorf, Styria, AT	5	24
Kemater Landmais	KL	Kematen, Tyrol, AT	6	24
Knillis	KN	Styria, AT	5	24
Krajova c29	KR	Craiova, Moravské Lieskové, SVK	7	24
Lacaune	LC	Lacaune, Languedoc-Roussillon-Midi-Pyrénées, FR	3	24
Lalin	LL	Lalín, Galicia, ES	2	24
Lucq de Bearn	LD	Lucq-de-Béarn, Aquitaine-Limousin-Poitou-Charentes, FR	3	24
Mahndorfer	MD	Northern Germany, DE	4	24
Maleksberger	MB	Northern Germany, DE	8	24
Millette du Lauragais 2	ML	Lauragais, Languedoc-Roussillon-Midi-Pyrénées, FR	3	24
Moncassin	MO	Moncassin, Languedoc-Roussillon-Midi-Pyrénées, FR	3	24
Nostrano dell Isola	ND	Northern Italy, IT	3	24
Oberhuber Martha	OM	Innsbruck, Tyrol, AT	1	24
Österreichische Landsorte	OE	Upper Austria, AT	9	24
Petkuser Ferdinand Rot	PE	Northeastern Germany, DE	10	24
Pfarrkirchner	PF	Pfarrkirchen, Bavaria, DE	4	24
Polnischer Landmais	PL	Poland, PL	8	24
Rheintaler Monsheim	RM	Monsheim, Rhineland-Palatinate, DE	8	24
Rheintaler St. Gallen	RT	St. Gallen, St. Gallen, CH	4	24
Rottaler	RO	Rottal-Inn, Bavaria, DE	3	24
Roux de Chalosse	RD	Chalosse, Aquitaine-Limousin-Poitou-Charentes, FR	3	24
Santiago	SA	Santiago de Compostela, Galicia, ES	2	23
Schindelmeiser	SC	Northeastern Germany, DE	4	23
Sornay	SO	Sornay, Bourgogne-Franche-Comté, FR	11	23
Strenzfelder	SF	Southeastern Germany, DE	4	23
Tremesino	TR	Mediterranean ES	2	24
Tui	TU	Tui, Galicia, ES	2	24
Viana	VI	Viana, Galicia, ES	2	24
Wantzenau	WA	La Wantzenau, Alsace-Champagne-Ardenne-Lorraine, FR	3	24

[a] Abbreviation.

[b] Abbreviations for European countries:

AT: Austria
CH: Switzerland
DE: Germany
ES: Spain
FR: France
IT: Italy
PL: Poland
SK: Slovakia

[c] The numbers denote the following institutions:

1 Research Centre for Agriculture and Forestry, Laimburg, Italy; obtained via Bavarian State Research Center, Freising, Germany
2 Spanish National Research Council (CSIC), Pontevedra / Zaragoza, Spain
3 Institut National de la Recherche Agronomique (INRA), Centre de Ressources Biologiques, Unité Experimental DiaScope, Mauguio, France
4 Universität Hohenheim, Institute of Plant Breeding, Seed Science and Population Genetics, Stuttgart, Germany
5 Saatzucht Gleisdorf, Gleisdorf, Austria; obtained via Bavarian State Research Center, Freising, Germany
6 Tyrolean Gene Bank, Innsbruck, Austria; obtained via Bavarian State Research Center, Freising, Germany
7 Crop Research Institute, Prague, Czech Republic; obtained via Bavarian State Research Center, Freising, Germany
8 Dow Agro Science LLC, München, Germany; obtained via Bavarian State Research Center, Freising, Germany
9 Bavarian State Research Center, Freising, Germany
10 Leibniz Institute of Plant Genetics and Crop Plant Research, Gatersleben, Germany; obtained via Bavarian State Research Center, Freising, Germany
11 Centre for Genetic Resources, Wageningen, Netherlands; obtained via Bavarian State Research Center, Freising, Germany
