# Supplementary material for: A comprehensive study of the genomic differentiation between temperate Dent and Flint maize
Source: Genome Biol. 2016 Jul 8;17:137. doi: 10.1186/s13059-016-1009-x (PMC4937532; doi:10.1186/s13059-016-1009-x)
Supplement: Additional file 2: Figure S1. — Metrics of the selection screens for 136 temperate inbred lines along the ten maize chromosomes based on genotyping data. (PDF 4242 kb) [file 13059_2016_1009_MOESM2_ESM.pdf]

Figure S1: Metrics of the selection screens for 136 temperate inbred lines along the ten maize chromosomes based on genotyping data. Dashed lines indicate the threshold per metric and windows exhibiting values below the respective threshold are shown in red for Dent and in blue for Flint. The positions of candidate genes are highlighted in the corresponding colors in the upper part of the F_{ST} plot with centromere positions shown as grey triangles in the lower part.

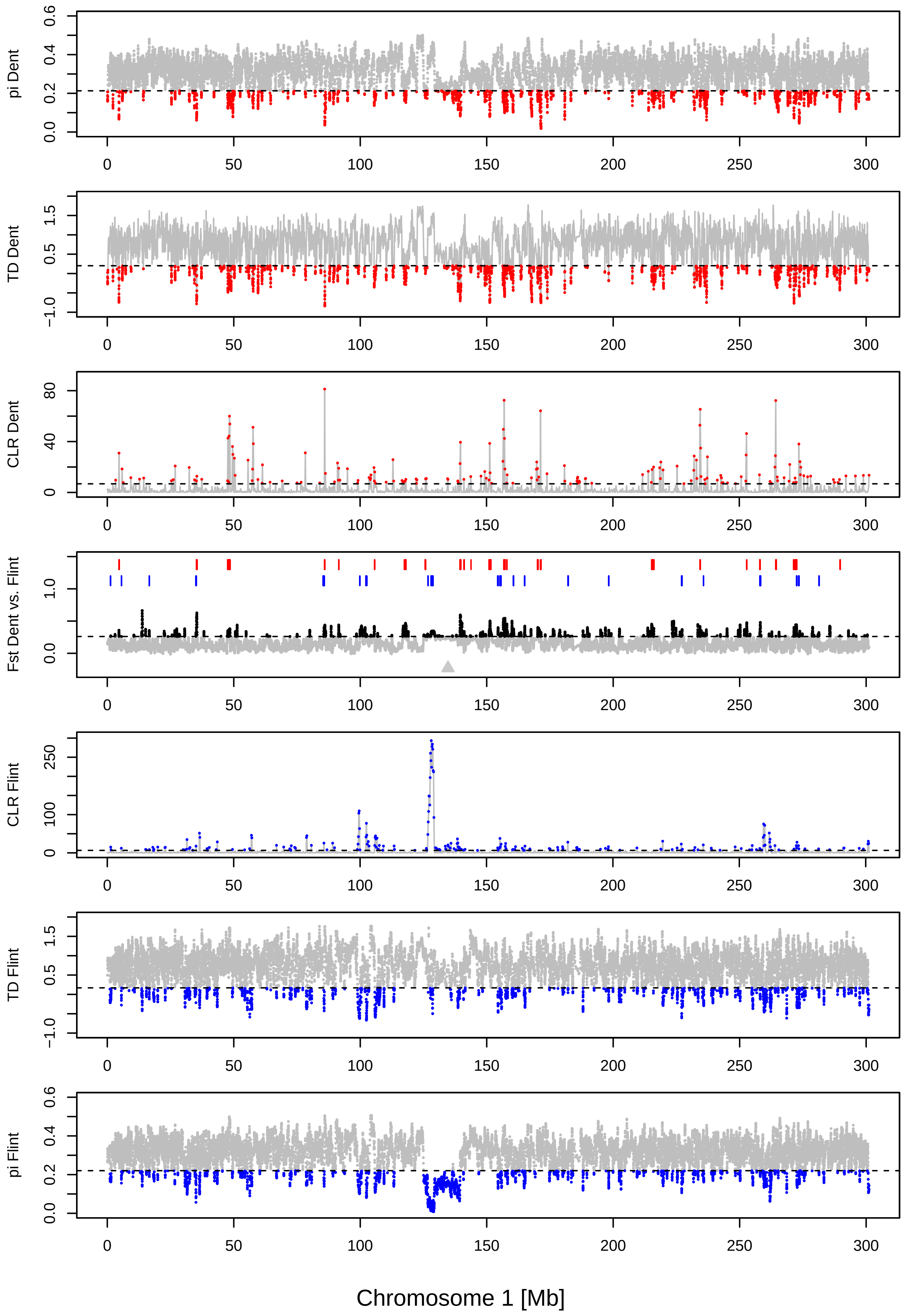
pi Dent
TD Dent
CLR Dent
Fst Dent vs. Flint
CLR Flint
TD Flint
pi Flint
0
50
100
150
200
250
300
0.0
0.2
0.4
0.6
−1.0
0.5
1.5
0
40
80
0.0
1.0
100
250
Chromosome 1 [Mb]

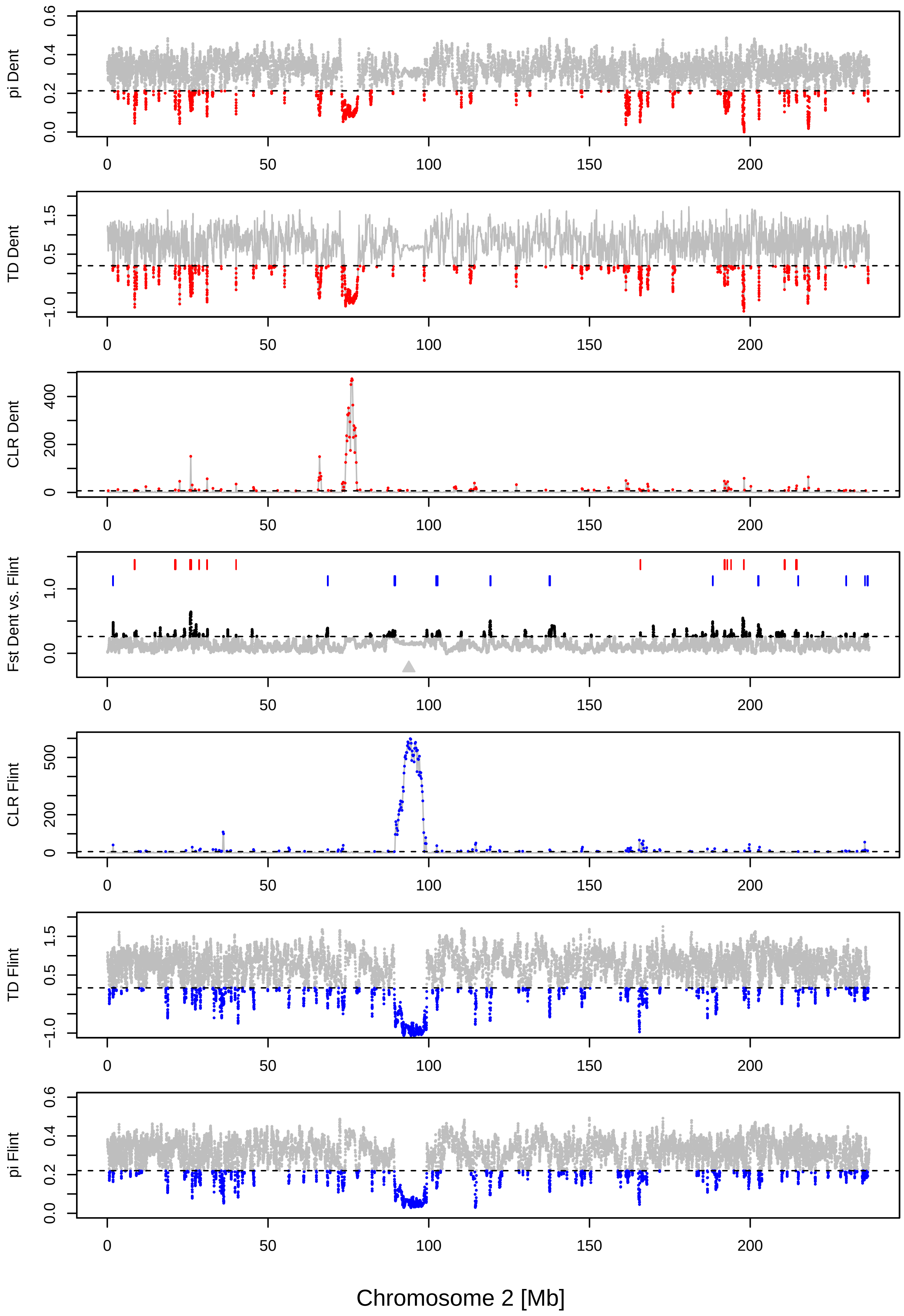

pi Dent
TD Dent
CLR Dent
Fst Dent vs. Flint
CLR Flint
TD Flint
pi Flint
0
50
100
150
200
Chromosome 2 [Mb]

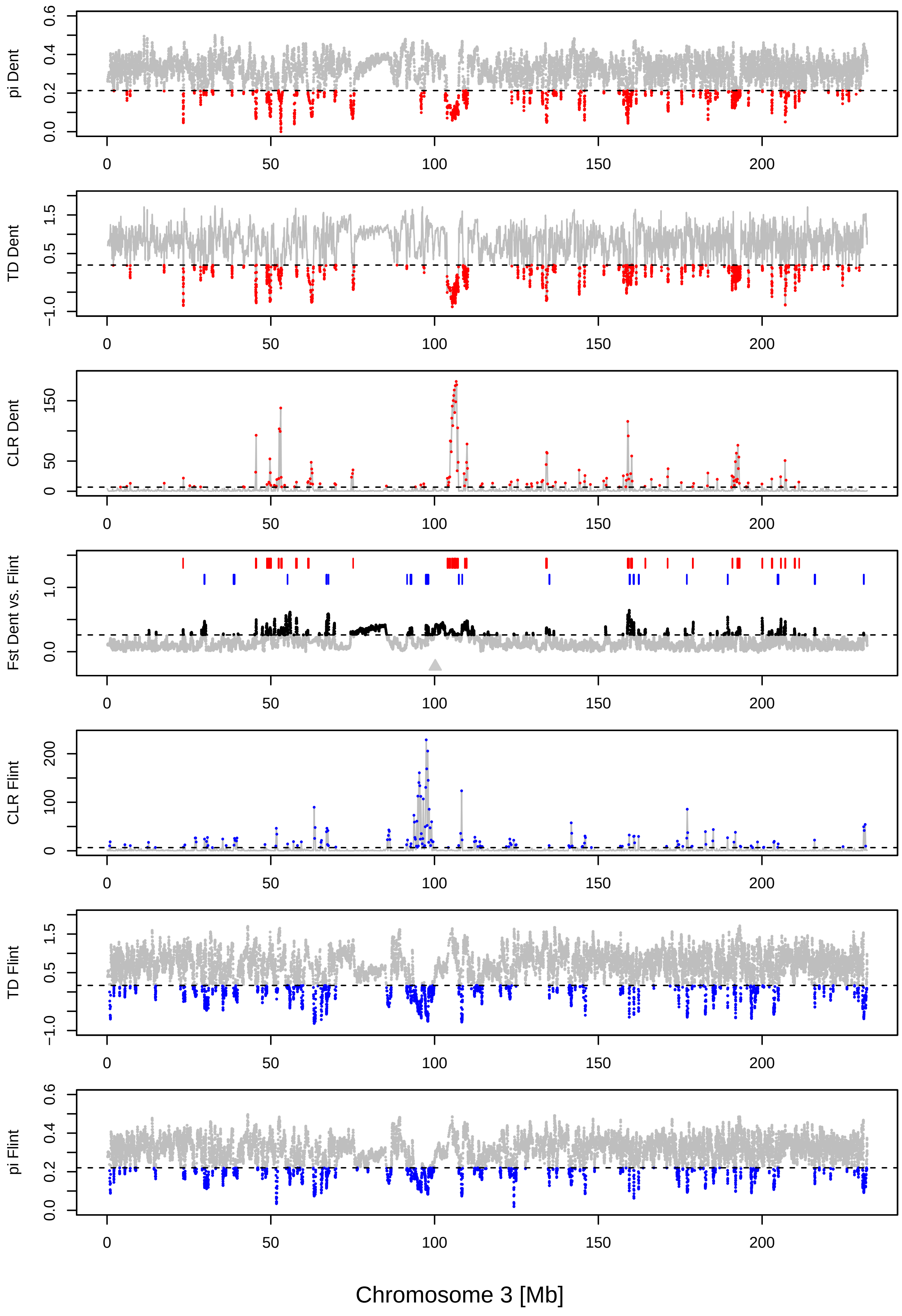

pi Dent
TD Dent
CLR Dent
Fst Dent vs. Flint
CLR Flint
TD Flint
pi Flint
Chromosome 3 [Mb]

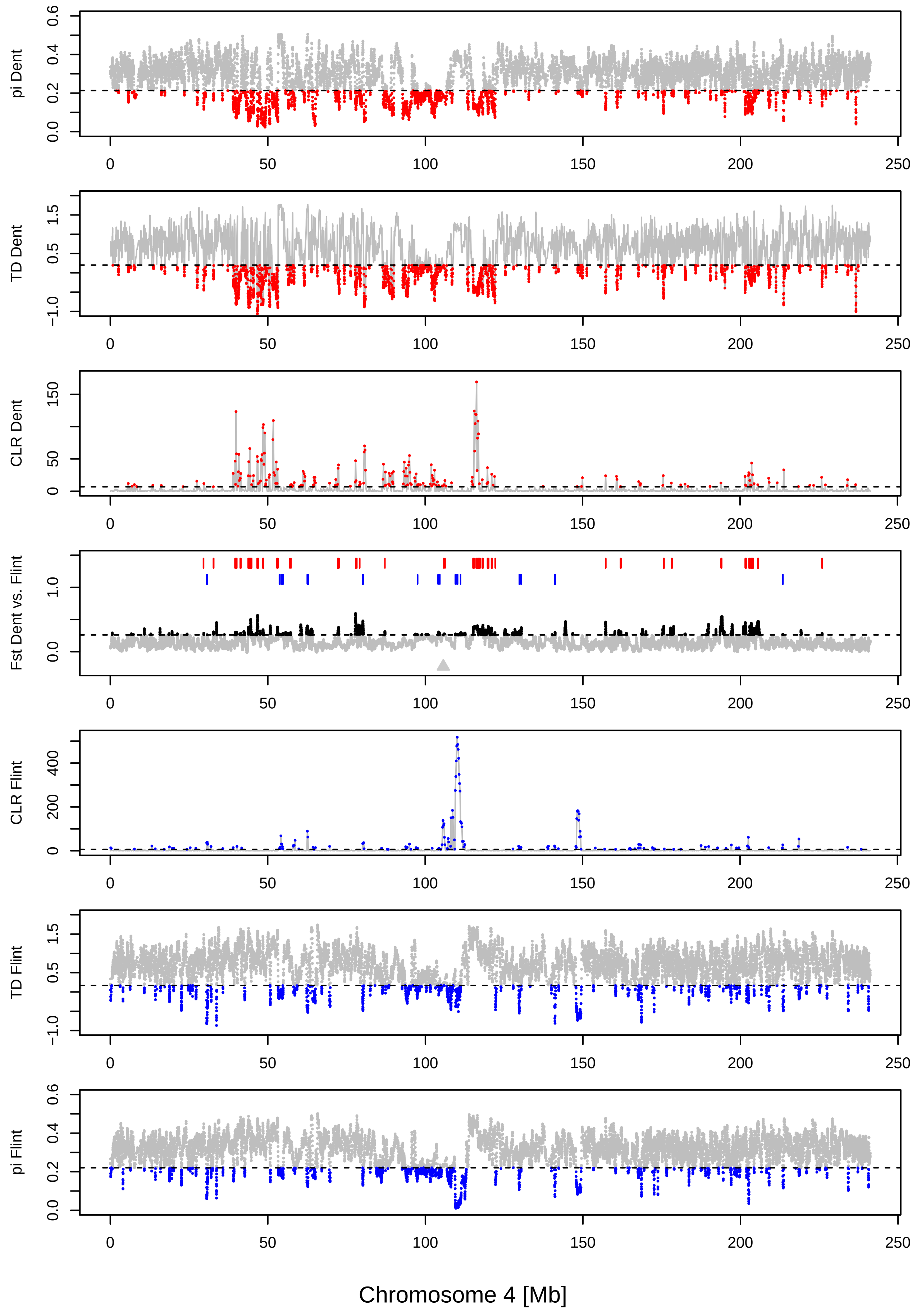

pi Dent
TD Dent
CLR Dent
Fst Dent vs. Flint
CLR Flint
TD Flint
pi Flint
0 50 100 150 200 250
Chromosome 4 [Mb]

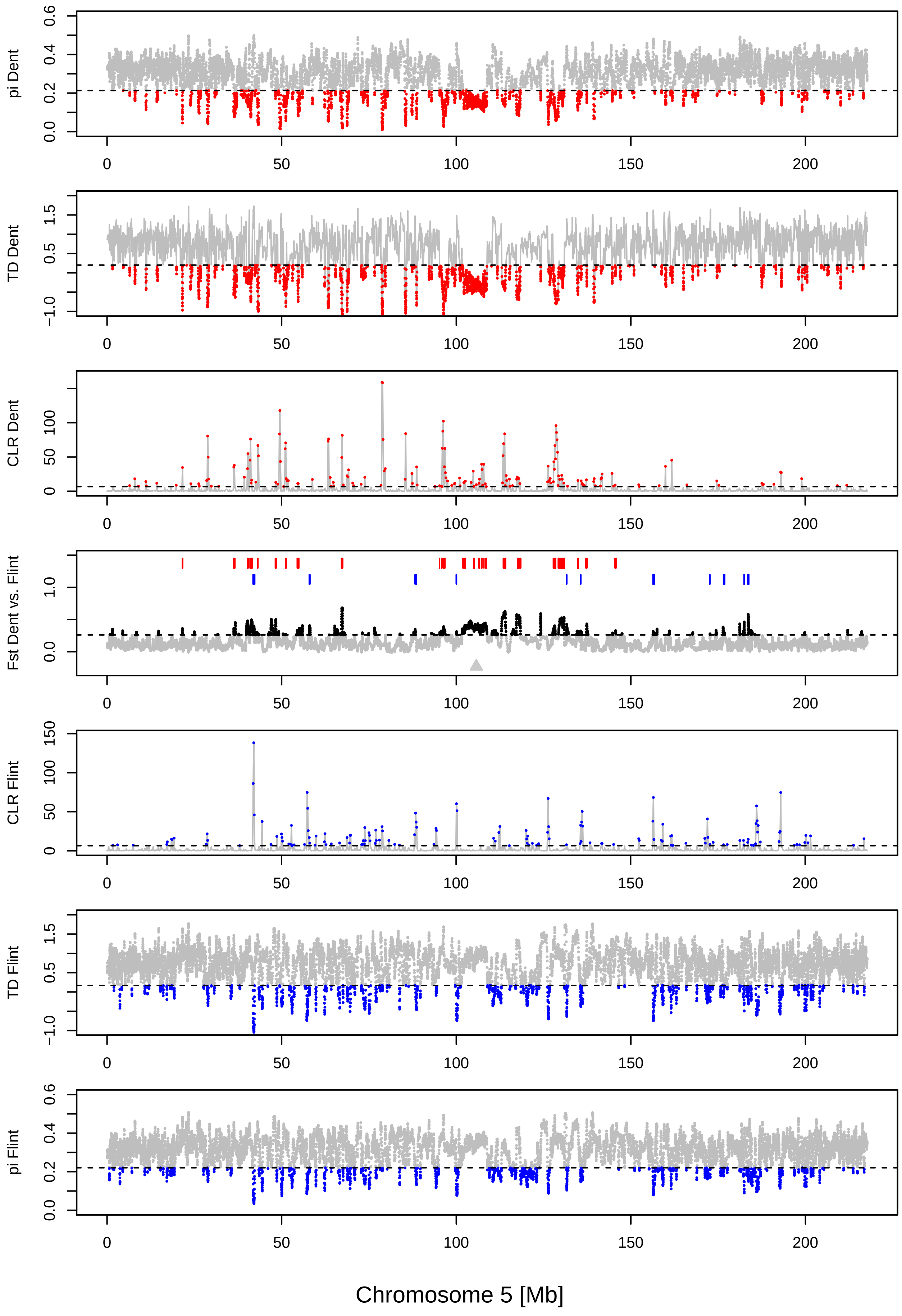
pi Dent
TD Dent
CLR Dent
Fst Dent vs. Flint
CLR Flint
TD Flint
pi Flint
Chromosome 5 [Mb]

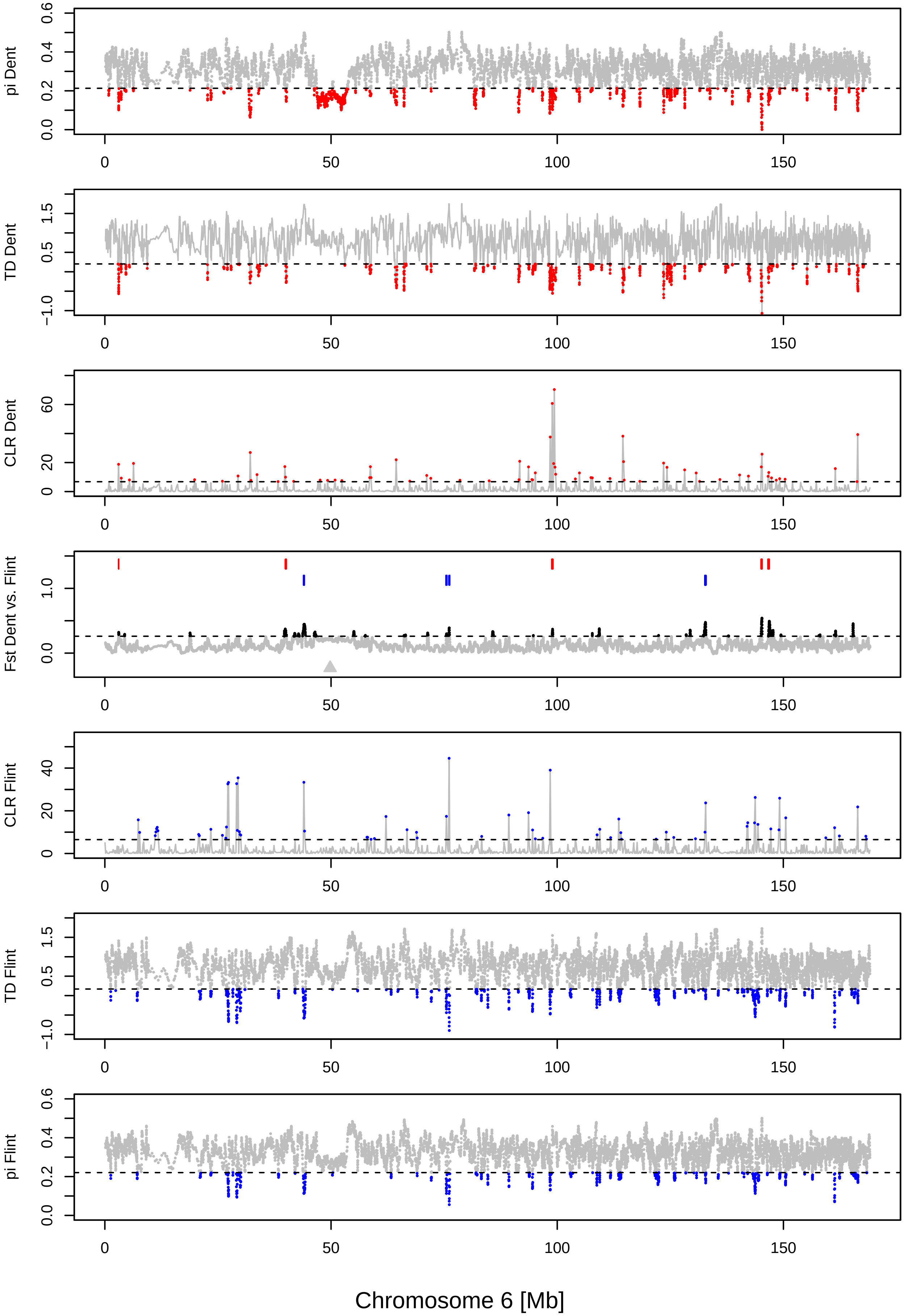

pi Dent
TD Dent
CLR Dent
Fst Dent vs. Flint
CLR Flint
TD Flint
pi Flint
0.0
0.2
0.4
0.6
−1.0
0.5
1.5
0
20
40
60
1.0
0
50
100
150
Chromosome 6 [Mb]

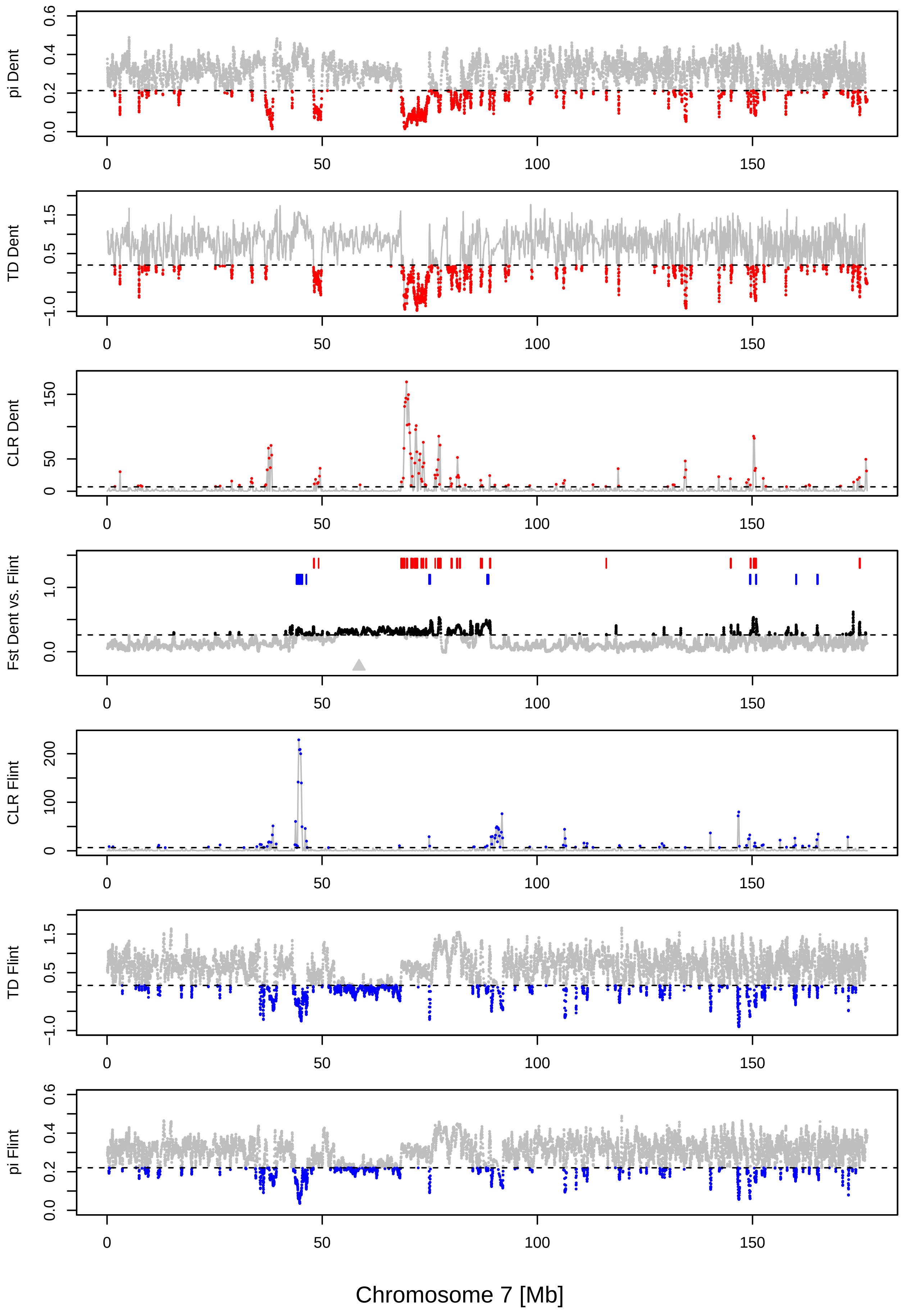

pi Dent
TD Dent
CLR Dent
Fst Dent vs. Flint
CLR Flint
TD Flint
pi Flint
Chromosome 7 [Mb]

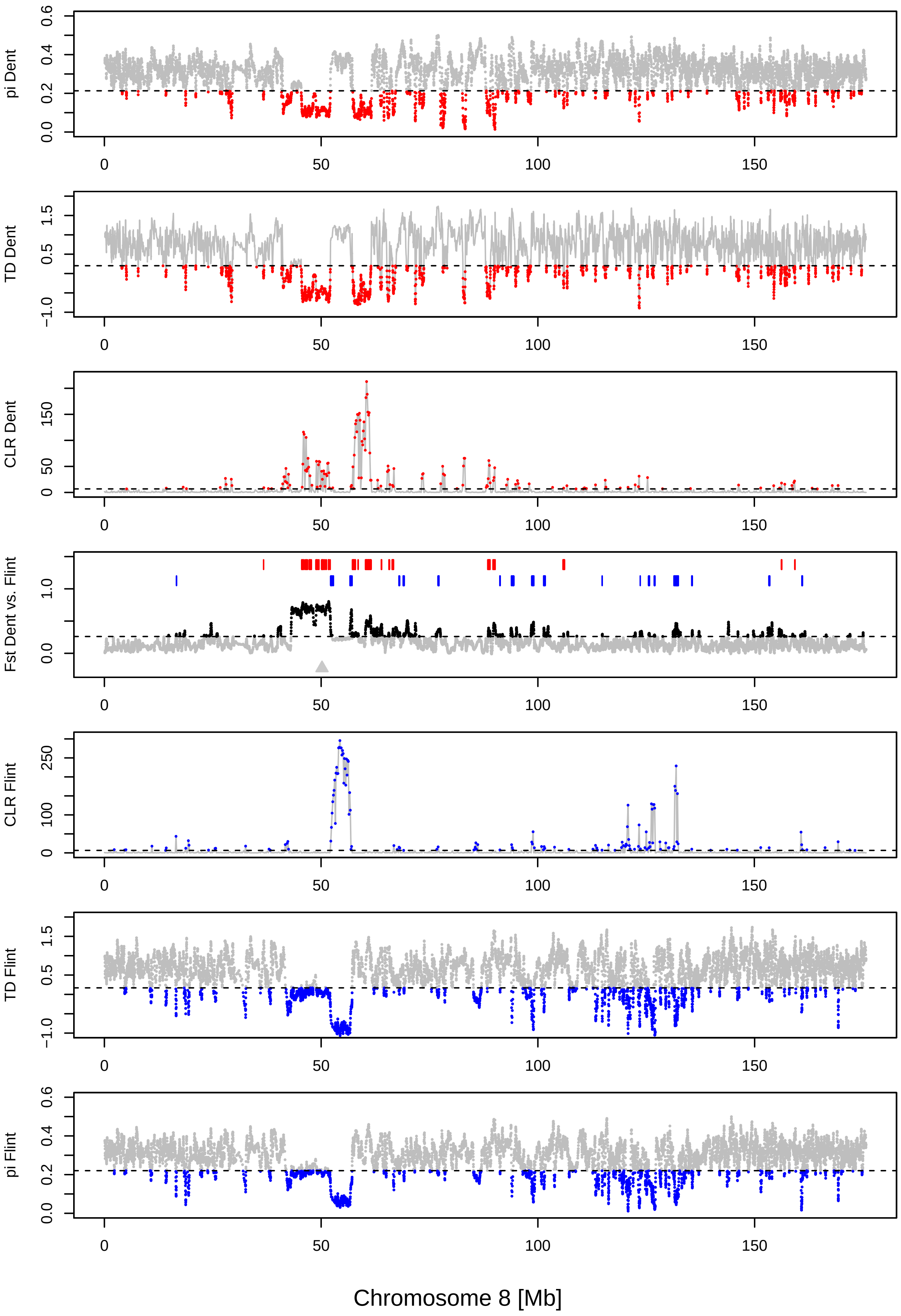
pi Dent
TD Dent
CLR Dent
Fst Dent vs. Flint
CLR Flint
TD Flint
pi Flint
0
50
100
150
Chromosome 8 [Mb]

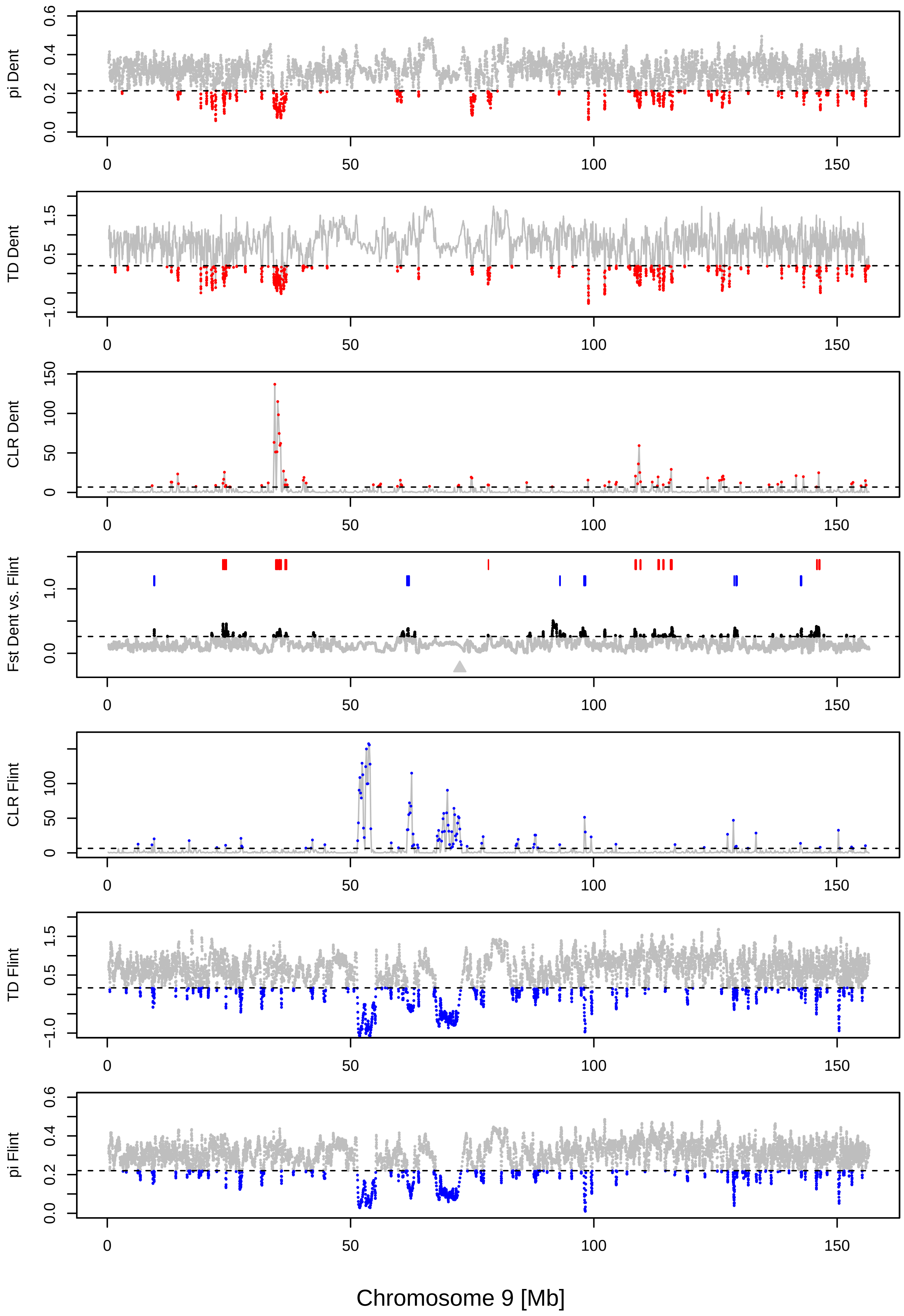
pi Dent
0.0
0.2
0.4
0.6
TD Dent
−1.0
0.5
1.5
CLR Dent
0
50
100
150
Fst Dent vs. Flint
0.0
1.0
CLR Flint
0
50
100
TD Flint
−1.0
0.5
1.5
pi Flint
0.0
0.2
0.4
0.6
0
50
100
150
Chromosome 9 [Mb]

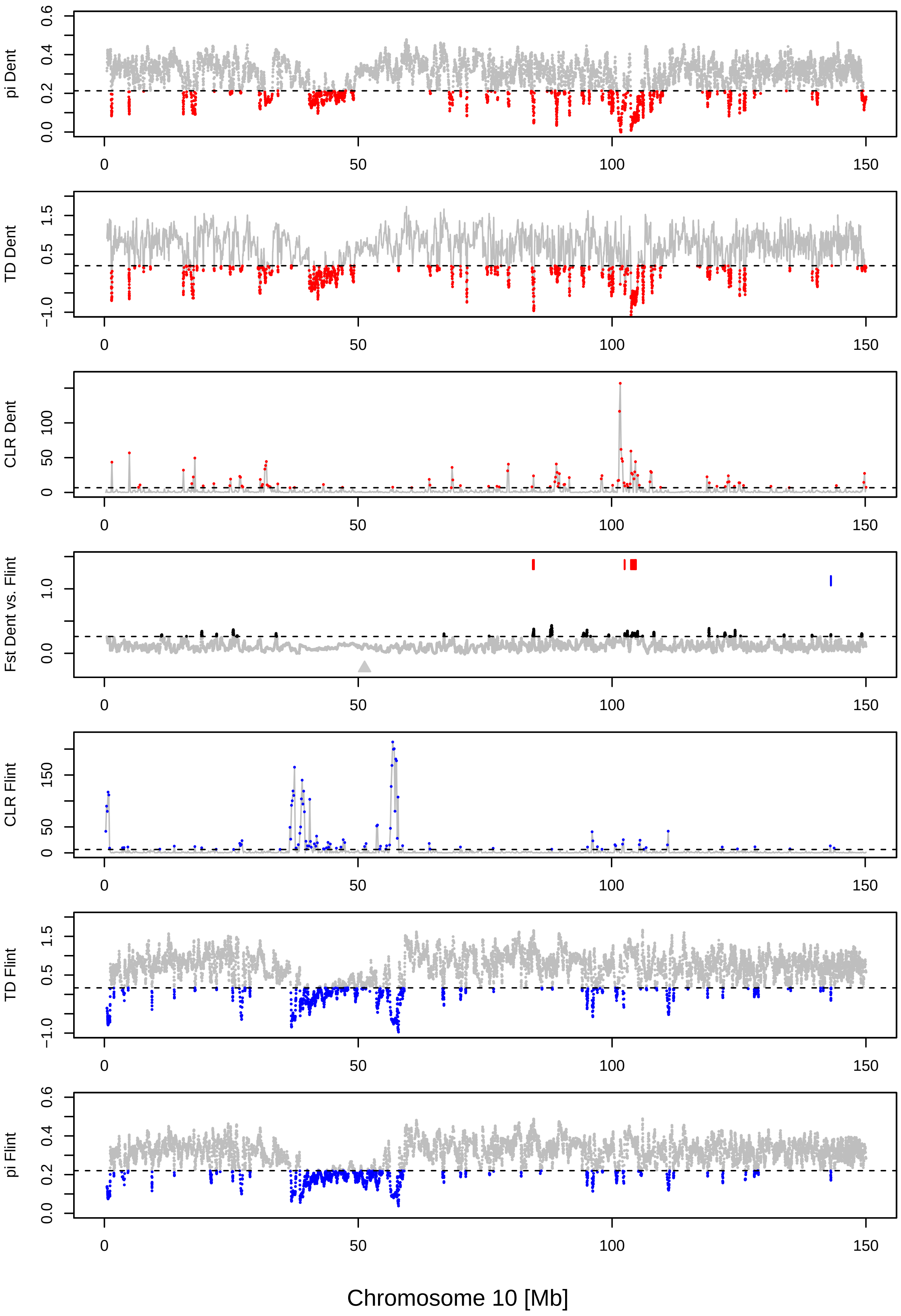
pi Dent
TD Dent
CLR Dent
Fst Dent vs. Flint
CLR Flint
TD Flint
pi Flint
Chromosome 10 [Mb]
